# Oxygen Defect Engineering Promotes Synergy Between Adsorbate Evolution and Single Lattice Oxygen Mechanisms of OER in Transition Metal‐Based (oxy)Hydroxide

**DOI:** 10.1002/advs.202303321

**Published:** 2023-10-09

**Authors:** Yu‐Han Wang, Lei Li, Jinghui Shi, Meng‐Yuan Xie, Jianhang Nie, Gui‐Fang Huang, Bo Li, Wangyu Hu, Anlian Pan, Wei‐Qing Huang

**Affiliations:** ^1^ Department of Applied Physics School of Physics and Electronics Hunan University Changsha 410082 P. R. China; ^2^ School of Materials Science and Engineering Hunan University Changsha 410082 P. R. China

**Keywords:** oxygen defects, adsorbate evolution mechanisms, lattice oxygen mechanisms, transition metal‐based (oxy)hydroxides, oxygen evolution reactions

## Abstract

The oxygen evolution reaction (OER) activity of transition metal (TM)‐based (oxy)hydroxide is dominated by the number and nature of surface active sites, which are generally considered to be TM atoms occupying less than half of surface sites, with most being inactive oxygen atoms. Herein, based on an in situ competing growth strategy of bimetallic ions and OH^−^ ions, a facile one‐step method is proposed to modulate oxygen defects in NiFe‐layered double hydroxide (NiFe‐LDH)/FeOOH heterostructure, which may trigger the single lattice oxygen mechanism (sLOM). Interestingly, by only varying the addition of H_2_O_2_, one can simultaneously regulate the concentration of oxygen defects, the valence of metal sites, and the ratio of components. The proper oxygen defects promote synergy between the adsorbate evolution mechanism (AEM, metal redox chemistry) and sLOM (oxygen redox chemistry) of OER in NiFe‐based (oxy)hydroxide, practically maximizing the use of surface TM and oxygen atoms as active sites. Consequently, the optimal NiFe‐LDH/FeOOH heterostructure outperforms the reported non‐noble OER catalysts in electrocatalytic activity, with an overpotential of 177 mV to deliver a current density of 20 mA cm^−2^ and high stability. The novel strategy exemplifies a facile and versatile approach to designing highly active TM‐LDH‐based OER electrocatalysts for energy and environmental applications.

## Graphical abstract

The proper oxygen defects promote the synergy between the adsorbate evolution mechanism (AEM) and single lattice oxygen mechanism (sLOM) of OER in NiFe‐based (oxy)hydroxide has been demonstrated, which can nearly maximize the use of surface metal and oxygen atoms as active sites, contributing ultralow overpotential of 177 mV (η_20_) and long‐term stability over 60 h, outmatching the reported non‐noble OER catalysts so far.

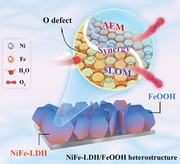

## 1 Introduction

One approach to sustainable hydrogen production is electrochemical water splitting, which includes the oxygen evolution reaction (OER) and hydrogen evolution reaction (HER). Due to repeated electron transfer, OER is more sluggish than HER and requires a high overpotential, above the minimum theoretical value of 1.23 V, which restricts its practical application.^[^ ^1^, ^2^, ^3^ ^]^ Noble metal‐based materials (e.g., Ir/IrO_2_) are considered as benchmark electrocatalysts for reducing the overpotential of OER,^[^ ^4^, ^5^, ^6^ ^]^ but are limited by their low economic benefit. Instead, earth‐abundant 3*d* transition metal (TM)‐based catalysts, such as TM‐based oxides or (oxy)hydroxides are demonstrated as promising alternatives for OER.^[^ ^7^, ^8^, ^9^, ^10^ ^]^

**Figure 1:**
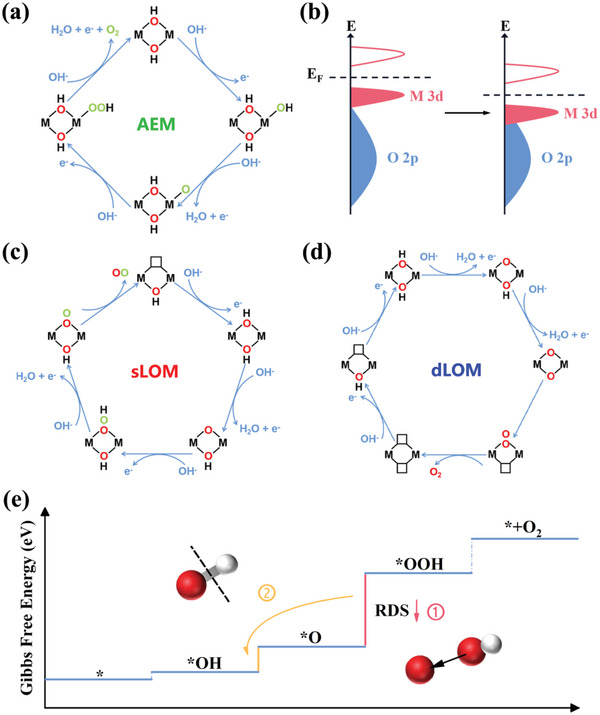
Schematic illustration of OER mechanisms and band diagrams. a) The schematic illustration of the adsorbate evolution mechanism (AEM), where the adsorbed oxygen and lattice oxygen are marked with green and red “O”, respectively. b) Schematic band structure diagrams showing the sinking of metal's *d*‐band energy into the *p*‐band of the oxygen ligand. c) The schematic illustration of a single lattice oxygen mechanism (sLOM). d) The schematic illustration of the double lattice oxygen mechanism (dLOM). e) Two strategies to improve OER performance are (1) lowering the barrier of RDS and (2) changing the RDS.

During the OER process, water oxidation typically occurs as a result of water molecules or hydroxide ions nucleophilic attacking metal sites, which is called as adsorbate evolution mechanism (AEM, **Figure** **1a**).^[^ ^11^, ^12^, ^13^ ^]^ High‐valent metal sites can help charge transfer by increasing the covalency between metal─oxygen (M─O) bonds, where the metal's *d*‐band energy sinks and even enters into the *p*‐band of the oxygen ligand (Figure 1b).^[^ ^14^, ^15^ ^]^ As a result, metal sites with higher valence states often have better catalytic activity.^[^ ^16^, ^17^, ^18^ ^]^ Interestingly, oxygen in the lattices of metal oxides and (oxy)hydroxides have recently been discovered to engage in surface reactions and play an important role in catalyst activity regulation, referred to as lattice oxygen mechanism (LOM, Figure 1c,d, Supporting Information).^[^ ^19^, ^20^, ^21^ ^]^ The activated lattice oxygen exhibits electrophilicity, promoting the subsequent nucleophilic attack of OH^−^; and it may also directly participate in the oxygen generation, lowering the reaction energy barrier in the OER process.^[^ ^22^, ^23^, ^24^ ^]^ For instance, lattice oxygen is proved to be involved in OER on the surface of pyrochlore oxides^[^ ^25^ ^]^ and Zn_0.2_Co_0.8_OOH. ^[^ ^24^ ^]^ The latest research indicates that both AEM and LOM could exist in OER process catalyzed by TM‐based (oxy)hydroxides, and they can even occur simultaneously.^[^ ^1^, ^26^ ^]^ However, the rate‐determining step (RDS) of AEM and LOM, which fundamentally hampers their electron transfer efficiency, is regarded as one of the barriers to the development of OER electrocatalysts.^[^ ^27^ ^]^ Ideally, the free energy barrier of each OER step should be tuned to approach the equilibrium potential of 1.23 eV,^[^ ^28^ ^]^ as evidenced by reducing the overpotential of the RDS or changing the RDS (Figure 1e). In practice, the overpotential of both two mechanisms could be typically lowered by modifying the electronic structure to obtain suitable M─O covalency.^[^ ^26^ ^]^

NiFe‐based (oxy)hydroxides, especially the layered double hydroxides (LDHs), are attractive OER catalysts due to their distinct layered structure and tunable electronic structure.^[^ ^8^, ^29^, ^30^, ^31^ ^]^ The surface TM atoms are generally considered to be active sites, which determine the OER activity of NiFe‐LDHs. To improve the OER activity of TM‐LDHs, previous studies have mainly focused on tuning the number and nature (particularly, electronic structure) of the surface TM atoms. For example, in previous works, some strategies have been proposed to optimize the electronic structure of TM atoms and enhance catalytic activity, i.e., doping of various cations,^[^ ^32^, ^33^ ^]^ optical triggering,^[^ ^27^ ^]^ and combining methods,^[^ ^34^, ^35^ ^]^ although the used methods are often complicated. However, the surface TM atoms only occupy less than half of surface sites, while most are inactive oxygen atoms. Therefore, how to maximize the use of surface sites as active sites, not just the TM atoms, is crucial but challenging in the development of superior TM‐based OER catalysts.

Here, we present a facile method for the one‐pot, one‐step hydrothermal synthesis of a hexagonal NiFe‐LDH/FeOOH heterostructure electrode with oxygen defects, based on the in situ competing growth strategy of bimetallic ions and OH^−^ ions. Only by tuning the amount of H_2_O_2_ addition, the oxygen defects can be effectively modulated. We reveal that oxygen defects can promote the nucleophilic attack of OH^−^ and the Fe─O sites synergy, i.e., the synergy between AEM and LOM, which could nearly maximize the use of surface TM and oxygen atoms as active sites to greatly improve the catalytic activity. Under alkaline conditions, the OER overpotentials of the optimized NiFe‐LDH/FeOOH at 20, 100, and 1000 mA cm^−2^ are only 177, 236, and 359 mV, respectively, with high stability, which is better than most of the NiFe‐based catalysts reported (Table S1, Supporting Information).^[^ ^34^, ^36^ ^]^ More importantly, differential electrochemical mass spectrometry (DEMS) measurements and first‐principle calculations reveal that the single lattice oxygen mechanism (sLOM) occurs during OER process, distinctly different from the double lattice oxygen mechanism (dLOM) in previous reports.

## 2 Results and Discussion

### 2.1 Synthesis and Characterization of NiFe‐LDH/FeOOH Heterostructure

**Figure 2:**
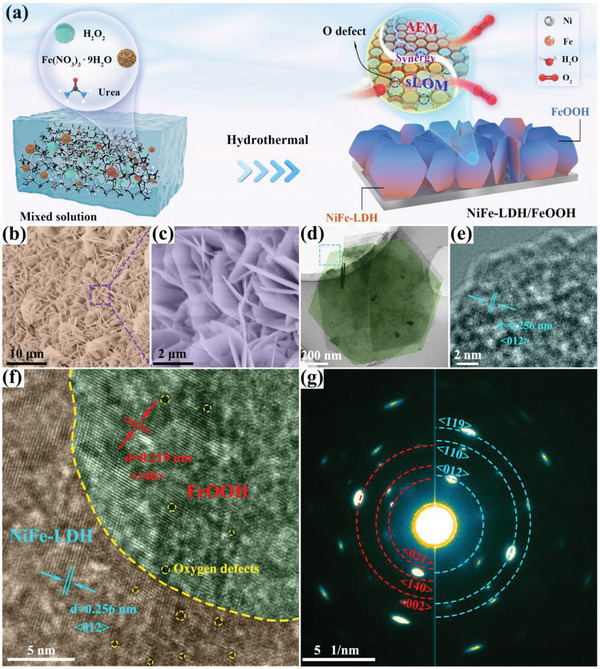
Synthesis and structural characterization of the NiFe‐LDH/FeOOH heterostructures. a) Schematic illustration of the synthesis of NiFe‐LDH/FeOOH heterostructures. A simple one‐step hydrothermal method to prepare NiFe‐LDH/FeOOH heterostructures, while the electronic structure can be tuned only by the addition of H_2_O_2_. b, c) Typical SEM images of NiFe‐LDH/FeOOH at different magnifications. d) TEM image of NiFe‐LDH/FeOOH. e, f) HRTEM images of NiFe‐LDH/FeOOH heterostructures at different magnifications, where the oxygen defects are marked with yellow dotted circles. g) SAED image of NiFe‐LDH/FeOOH heterostructures.

The fabrication procedure of the hexagonal NiFe‐LDH/FeOOH heterostructure is depicted in **Figure** **2**a. Via a facile competing growth strategy of bimetallic ions with OH^−^ ions, the NiFe‐LDH/FeOOH heterostructure was in situ grown on nickel foam (NF) through one‐pot hydrothermal treatment (Figure S1, Supporting Information). Scanning electron microscopy (SEM) images (Figure 2b,c) reveal that the uniformly dense 2D nanosheets are staggered into a 3D open architecture on the NF. The morphology and structure of NiFe‐LDH/FeOOH heterostructure are further characterized by transmission electron microscopy (TEM) in detail. TEM images clearly confirm the 2D hexagonal nanosheet‐like characteristics of the NiFe‐LDH/FeOOH with a side length of ≈ 500 nm and the transparency of the nanosheets indicates the ultrathin nature (Figure 2d and Figure S2, Supporting Information). The high‐resolution TEM (HRTEM) images of NiFe‐LDH/FeOOH (Figure 2e,f) obviously reveal the edge and visible interface of the hexagonal nanosheet, illustrating that FeOOH is embedded with NiFe‐LDH. The intimate interfacial contact (Figure 2f) between NiFe‐LDH and FeOOH suggests the successful construction of a bimetallic hydroxide heterostructure. The well‐resolved lattice fringes with an interplanar spacing of 0.256 nm in the red area and 0.219 nm in the green area can be assigned to the (012) plane of NiFe‐LDH and (140) plane of FeOOH, respectively (Figure S3, Supporting Information). Besides, the selected region electron diffraction (SAED) diagram (Figure 2g) shows blue bright rings corresponding to the (012), (110), and (119) diffraction planes of the NiFe‐LDH phase and red bright rings referring to the (002), (140), and (021) diffraction planes of the FeOOH phase. The existence of Ni, Fe, and O elements alongside their homogenous distribution throughout the whole heterostructure is also demonstrated by elemental mapping (Figure S4, Supporting Information), further verifying the successful synthesis of NiFe‐LDH/FeOOH heterostructure. In contrast, bare Ni(OH)_2_ synthesized under similar experimental conditions in the absence of Fe(NO_3_)_3_ is comprised of small irregular nanosheets agglomerated randomly (Figure S5, Supporting Information), indicating the pivotal role of the competing interaction between bimetallic ions in the formation of hexagonal nanosheets.

### 2.2 Growth Mechanism and Regulation of NiFe‐LDH/FeOOH Heterostructure

**Figure 3:**
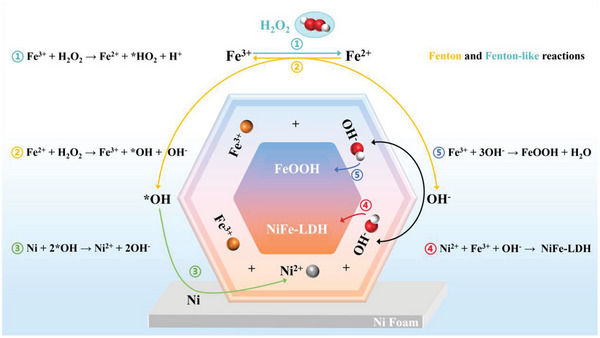
Schematic illustration of the growth mechanism of NiFe‐LDH/FeOOH heterostructures when adding H_2_O_2_. Based on Fenton and Fenton‐like reactions to produce *OH and OH^−^, the growth of NiFe‐LDH and FeOOH can thus be modulated by H_2_O_2_.

Based on the experimental results, the growth mechanism of hexagonal NiFe‐LDH/FeOOH heterostructures could be derived below, as shown in **Figure** **3** and Supporting Information. With an increase of H_2_O_2_ addition in the reaction solution, Fenton‐like and Fenton reactions will become more intense and produce large amounts of OH^−^ ions, which accelerate the precipitation with Ni^2+^ and Fe^3+^ ions. Notably, the competition precipitation of bimetallic ions with OH^−^ ions leads to the formation of uniform large NiFe‐LDH/FeOOH hexagonal nanosheets that interlace with each other forming a 3D open architecture. Therefore, we can readily tune the ratio of FeOOH to NiFe‐LDH in the NiFe‐LDH/FeOOH heterostructure by *only* varying the amount of H_2_O_2_. Moreover, the difference in the concentration of Ni^2+^ and Fe^3+^ surrounding the NF and their different coordination numbers influence the chemical environment leading to the generation of oxygen defects.^[^ ^37^ ^]^ This competitive growth can tune not only the component ratio of the heterostructure but also the concentration of oxygen vacancy, thus enhancing the OER performance of NiFe‐LDH/FeOOH heterostructure, as discussed in the next sections.

**Figure 4:**
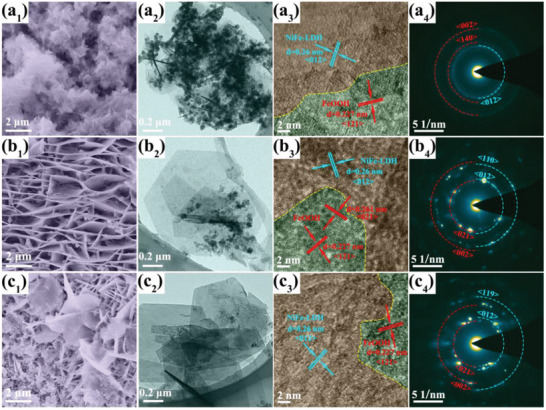
Structural characterization of NiFe‐LDH/FeOOH with a) 0 mL, b) 0.5 mL, and c) 2 mL H_2_O_2_. a_1_, b_1_, c_1_) Typical SEM images. a_2_, b_2_, c_2_) TEM images. a_3_, b_3_, c_3_) HRTEM images. a_4_, b_4_, c_4_) SAED images.

Structural characterization is conducted to investigate the effect of H_2_O_2_ on the morphology and structure of NiFe‐LDH/FeOOH. SEM images (Figure 2a,b, and **Figure** **4**a_1_,b_1_,c_1_) clearly reveal that all the synthesized NiFe‐LDH/FeOOH nanosheets grow vertically on NF, generating an interconnected network with enhanced mechanical stability. The sizes of these nanosheets gradually grow in proportion to the amount of H_2_O_2_ added, whereas many small irregular nanosheets appear besides hexagonal nanosheets when the H_2_O_2_ addition reaches 2 mL. TEM images (Figure 2c, and Figure 4a_2_,b_2_,c_2_) further illustrate the similar structural changes of NiFe‐LDH/FeOOH nanosheets. With the addition of H_2_O_2_, the small fragmented nanosheets become less and expand into larger hexagonal nanosheets, indicating that H_2_O_2_ can promote the growth of NiFe‐LDH/FeOOH hexagonal nanosheets. The HRTEM images (Figure 2d,e, and Figure 4a_3_,b_3_,c_3_) clearly show the lattice fringes with distance of 0.26 nm corresponding to the (012) plane of NiFe‐LDH (red area), and the lattice fringes of 0.227 nm and 0.261 nm assigning to the (121) and (021) plane of FeOOH (green area), respectively. Moreover, along with the addition of H_2_O_2_, SAED images (Figure 2j, and Figure 4a_4_,b_4_,c_4_) reveal the variation of NiFe‐LDH and FeOOH. The SAED diagrams of NiFe‐LDH and FeOOH are composed of bright rings marked with blue and red dotted lines, respectively. Compared with the H_2_O_2_‐free sample (Figure 4a_4_), the brightness of the diffraction diagrams of the H_2_O_2_‐added sample is significantly enhanced, suggesting that adding H_2_O_2_ favors the growth of NiFe‐LDH/FeOOH and improves the crystallinity of the samples. Besides, the atomic ratios of Ni, Fe, O, and C elements in distinct samples are determined using an energy dispersive spectrometer (EDS) (Figure S6, Supporting Information). In the H_2_O_2_‐free sample, the atomic ratios of Ni and Fe are 9.13% and 5.74%, respectively, indicating that the weak reaction only produces a tiny quantity of NiFe‐LDH in NiFe‐LDH/FeOOH. With 0.5 mL H_2_O_2_ addition, the atomic ratios of Ni and Fe significantly rise, suggesting the ratio of NiFe‐LDH increases; as the amount of H_2_O_2_ is increased to 1 mL, the ratio of Ni and Fe almost remains unchanged, indicating the continued growth of NiFe‐LDH/FeOOH. However, 2 mL H_2_O_2_ addition increases the atomic ratio of Fe relatively, revealing more FeOOH is generated.

**Figure 5:**
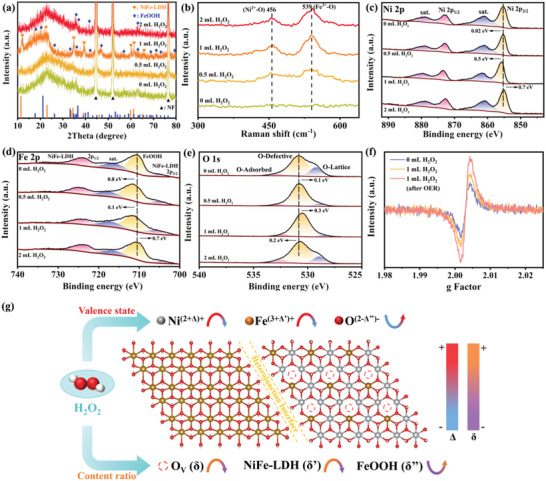
The texture properties and chemical structures characterization of NiFe‐LDH/FeOOH with 0, 0.5, 1, and 2 mL H_2_O_2_. a) XRD patterns of the samples. b) Raman spectrum of the samples. c–e) High‐resolution XPS spectra of Ni 2p, Fe 2p, and O 1s of the samples. f) EPR curves of the samples. g) Schematic illustration of the electronic structure and valence state variation of NiFe‐LDH/FeOOH with the influence of H_2_O_2_, where the Δ and δ represent the change amount of valence state and content ratio, respectively.

To further study the structure variations with the addition of H_2_O_2_, XRD is performed on the synthesized NiFe‐LDH/FeOOH heterostructures, as presented in **Figure** **5**a. All the XRD patterns of NiFe‐LDH/FeOOH feature the characteristic peaks of FeOOH (PDF#29‐0713) and NiFe‐LDH (PDF#40‐0215), confirming the co‐growth of NiFe‐LDH and FeOOH. Notably, the intensity of diffraction peaks alters dramatically as H_2_O_2_ addition increases. As the addition of H_2_O_2_ increases from 0 mL to 1 mL, the diffraction peaks of NiFe‐LDH and FeOOH steadily grow, while the peaks of NiFe‐LDH increase more obviously than that of FeOOH, indicating that the addition of H_2_O_2_ accelerates the etching of Ni foam and more Ni^2+^ ions produced participates in the formation of NiFe‐LDH. Nonetheless, further increasing the H_2_O_2_ addition results in an obvious peak enhancement of FeOOH compared with that of NiFe‐LDH, which can be ascribed to the large quantity of FeOOH formed. Raman spectroscopy is also performed to further confirm the component of as‐prepared NiFe‐LDH/FeOOH (Figure 5b). The peaks of Raman shift at ≈ 456 and 539 cm^−1^ correspond to Ni^2+^─O and Fe^3+^─O in NiFe‐LDH, respectively.^[^ ^37^, ^38^ ^]^ Both peaks increase along with the H_2_O_2_ addition since H_2_O_2_ boosts the formation of hydroxyl radicals (*OH) and OH^−^ ions and produces a large amount of NiFe‐LDH owing to the Fenton‐like and Fenton reactions.

XPS analysis is carried out to further study the elemental composition, surface chemical states, and the associated interaction between atoms of NiFe‐LDH/FeOOH. The XPS survey spectrum in Figure S7 (Supporting Information) reveals the coexistence of Ni, Fe, and O elements in NiFe‐LDH/FeOOH. The high‐resolution Ni 2p spectrum (Figure 5c) displays two major peaks, Ni 2p_3/2_ and Ni 2p_1/2_, which can be fitted into four sub‐peaks. The peaks of Ni 2p_3/2_ at 861.6 and 855.8 eV can be attributed to the satellites and Ni^2+^ in NiFe‐LDH, respectively. Similarly, the Ni 2p_1/2_ peaks at 879.5 and 873.5 eV represent satellites and Ni^2+^ in NiFe‐LDH, respectively.^[^ ^40^, ^41^ ^]^ Besides, four peaks are observed in the Fe 2p spectrum (Figure 5d), where peaks at 705.7 and 724.4 eV are ascribed to Fe 2p_3/2_ and Fe 2p_1/2_ of Fe^3+^ in NiFe‐LDH, and peaks at 711.8 and 717.9 eV are assigned to FeOOH phase and satellites, respectively.^[^ ^39^ ^]^ With the increase of H_2_O_2_ addition, the variation of peak area reflects that the content ratio of NiFe‐LDH increases and then decreases, while the opposite is true for FeOOH in NiFe‐LDH/FeOOH (Figure S8, Supporting Information), which is consistent with above XRD results. Furthermore, the O 1s spectrum (Figure 5e) exhibits three major peaks at 529.1, 530.5, and 532.3 eV, classified as lattice oxygen (O‐Lattice), defective oxygen (O‐Defective), and oxygen‐containing functional groups adsorbed on the surface (O‐Adsorbed).^[^ ^42^, ^43^ ^]^ It is worth mentioning that both Ni 2p peaks and Fe 2p peaks shift to the higher binding energy and then to the lower binding energy, while the O 1s peaks shift in the opposite directions to remain electrically neutral with the addition of H_2_O_2_. The peak shift of Ni 2p and Fe 2p can be caused by the alteration of metal valence. The elevated metal valence can promote lattice oxygen activation and produce oxygen defects,^[^ ^14^ ^]^ resulting in the decrease of the peak area ratio of the O‐Lattice and the increase of the peak area ratio of O‐Defective. Previous studies have shown that oxygen activity may be indirectly evaluated by characterizing the metal oxidation state, and a higher oxidation state of the transition metal is typically associated with increased oxygen activity.^[^ ^44^, ^45^ ^]^ Significantly, when the H_2_O_2_ level is 1 mL, the binding energies and valence of Ni 2p and Fe 2p in the obtained NiFe‐LDH/FeOOH reach the highest, while those of O 1s become the lowest, and the peak area ratio of O‐Defective is the highest, suggesting both the elevated metal valence and the content ratio of oxygen defects to the maximum, which helps enhance the OER performance.

Subsequently, the evolution of oxygen vacancies in the prepared samples was performed by electron paramagnetic resonance (EPR) measurement (Figure 5f). The EPR curve of NiFe‐LDH/FeOOH synthesized with 1 mL H_2_O_2_ exhibited a higher intensity than that of the H_2_O_2_‐free sample, demonstrating the higher oxygen vacancy concentration. The EPR results further confirmed the successful regulation and variation of oxygen vacancies, which is consistent with the XPS analysis. The aforementioned results suggest that the addition of H_2_O_2_ can effectively adjust the valence state and surface electrical structure of NiFe‐LDH/FeOOH nanosheets, which is illustrated in Figure 5g. The elevation of metal valence states and oxygen defects induced^[^ ^32^ ^]^ would enhance electrophilicity, promote the subsequent nucleophilic attack of OH^−^, and thus improve the catalytic activity of OER.^[^ ^44^ ^]^ However, further increasing the addition of H_2_O_2_ results in more FeOOH produced which covers the NiFe‐LDH nanosheet. The low intrinsic activity and electrical conductivity of FeOOH would reduce the apparent activity of NiFe‐LDH/FeOOH.^[^ ^30^ ^]^

### 2.3 Electrocatalytic Activity and Stability toward the OER

**Figure 6:**
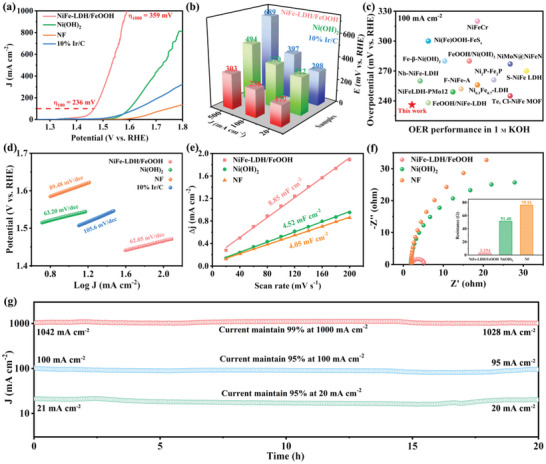
Electrochemical characterization of NiFe‐LDH/FeOOH heterostructures. a) Linear sweep voltammetry (LSV) curves recorded at a scan rate of 2 mV s^−1^ in 1.0 M KOH. b) Overpotential comparison of above samples at the current densities of 20, 100, and 500 mA cm^−2^. c) Electrocatalytic OER activity for NiFe‐LDH/FeOOH in comparison with other catalysts on NF. d) Tafel plots of NiFe‐LDH/FeOOH, Ni(OH)_2_, NF, and 10% Ir/C electrodes. e) The double‐layer capacitance (*C*_dl_) for NiFe‐LDH/FeOOH, Ni(OH)_2_, and NF electrodes derived from the CV plots. f) Electrochemical impedance spectroscopy (EIS) Nyquist plots for these electrodes. Inset: The value of *R*_ct_. g) Time‐dependent OER performance for NiFe‐LDH/FeOOH electrode at current densities of 20, 100, and 1000 mA cm^−2^ for 20 h.

Herein, the OER performance of synthesized electrodes is tested by linear sweep voltammetry (LSV) curves (Figures S9 and S10, Supporting Information). It can be observed that all the NiFe‐LDH/FeOOH samples show great electrocatalytic OER activity, which first increases and then decreases for the sample synthesized with increasing H_2_O_2_. This activity variation would be attributed to the effective modulation of oxygen defects in NiFe‐LDH/FeOOH, as demonstrated in the previous section. NiFe‐LDH/FeOOH synthesized with 1 mL H_2_O_2_ addition shows the smallest overpotential at the same current density (Figure S9a, Supporting Information), the lowest Tafel slope (Figure S9b, Supporting Information), and the highest stability (Figure S10, Supporting Information) among the four samples, indicating its superior catalytic activity. Then, the OER performance of NiFe‐LDH/FeOOH with 1 mL H_2_O_2_ is compared with those of bare Ni(OH)_2_, NF, and commercial 10% Ir/C. The LSV curves of the above samples are shown in **Figure** **6**a, while Figure 6b displays the overpotentials of these samples at current densities of 20, 100, and 500 mA cm^−2^. Among all samples, NiFe‐LDH/FeOOH requires only 177 mV overpotential to achieve a current density of 20 mA cm^−2^, which is significantly lower than the values of bare Ni(OH)_2_ (322 mV), NF (401 mV), and 10% Ir/C (298 mV). Of note, only 359 mV overpotential is needed for NiFe‐LDH/FeOOH to deliver the large current density of 1000 mA cm^−2^ (Figure 6a), satisfying the required large current density for industrial water splitting. Compared with the reported TM‐based catalysts, NiFe‐LDH/FeOOH heterostructure has a much lower overpotential, demonstrating its outmatching OER performance in alkaline circumstances (Figure 6c and Table S1, Supporting Information). Subsequently, the apparent kinetics of catalysts are evaluated by the Tafel slope utilizing the LSV curves (Figure 6d). NiFe‐LDH/FeOOH heterostructure displays a Tafel slope of 62.05 mV dec^−1^, smaller than those of bare Ni(OH)_2_, NF, and 10% Ir/C as depicted, illustrating the stronger reaction kinetics over NiFe‐LDH/FeOOH. To obtain a more accurate Tafel slope, the Tafel slope is also calculated using the staircase cyclic voltammetry (SCV) test (Figure S11, Supporting Information). The Tafel slopes calculated by LSV and SCV are close, which further strengthens the accuracy of the Tafel slope.

To gain insight into the mechanism of the high OER activity of NiFe‐LDH/FeOOH, the double‐layer capacitance (C_dl_) is measured to examine the electroactive sites of the catalysts. In the absence of a redox process, CV measurements (Figure S12, Supporting Information) are carried out at various scan rates in the potential range of the non‐Faradaic zone. The calculated C_dl_ of NiFe‐LDH/FeOOH heterostructure is 8.85 mF cm^−2^, higher than those of Ni(OH)_2_ (4.52 mF cm^−2^) and NF (4.05 mF cm^−2^), suggesting that NiFe‐LDH/FeOOH possesses more active sites for catalytic reactions (Figure 6e). This higher C_dl_ value of NiFe‐LDH/FeOOH is due to its 3D open construction, which is made up of thin 2D hexagonal nanosheets with large surface area and more active sites exposed to the reaction, favoring superior OER catalytic activity. As a complement, electrochemical impedance spectroscopy (EIS) is adopted to assess the intrinsic kinetics of catalysts. For further understanding of the reaction kinetics, the Nyquist plots are modeled using an equivalent circuit. Figure 6f shows the corresponding Nyquist plots of Ni(OH)_2_, NF, and NiFe‐LDH/FeOOH. Subsequently, these curves are fitted into a simple equivalent circuit (Figure S13, Supporting Information) to calculate the charge transfer resistance (R_ct_). As displayed in the inset of Figure 6f, the R_ct_ of NiFe‐LDH/FeOOH (3.254 Ω) is much smaller than those of Ni(OH)_2_ (51.40 Ω) and NF (75.91 Ω). The lower R_ct_ value means faster electron transfer, resulting in lower overpotential, which is consistent with the LSV results.

**Figure 7:**
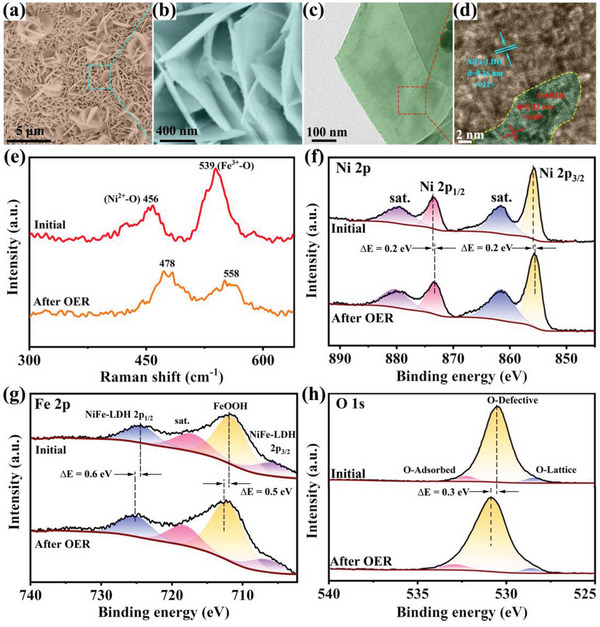
Structural and chemical structure characterization of the NiFe‐LDH/FeOOH heterostructures after stability test. a,b) Typical SEM images with different magnifications. c) TEM image. d) HRTEM image. e) Raman spectrum. f–h) High‐resolution XPS spectra of Ni 2p, Fe 2p, and O 1s spectra, respectively.

Aside from the superior OER activity, catalytic stability is another important criterion for practical application. The stability test is performed by applying chronoamperometric electrolysis in 1.0 M KOH. The chronoamperometric curves in Figure 6g indicate that after 20 hours of testing, merely 2∼5% of the current density loss is observed, revealing the satisfactory electrochemical stability of the NiFe‐LDH/FeOOH electrode. Meanwhile, the SEM images (**Figure** **7**a,b) reveal that NiFe‐LDH/FeOOH after the stability test still maintains the 3D open structure composed of vertically arranged 2D hexagonal nanosheets, comparable to the initial morphology. Figure 7c demonstrates the TEM image of NiFe‐LDH/FeOOH after the stability test, indicating similar morphology and structure to the initial sample. The HRTEM image (Figure 7d) reveals the well‐defined lattice fringes with spacings of 0.26 nm, which correspond to the (012) facet of orthorhombic NiFe‐LDH, while lattice fringes with spacings of 0.22 nm are matched with the (140) facet of FeOOH. In addition, blue bright rings indexed to the (110) and (012) diffraction planes of NiFe‐LDH, and red bright rings respectively representing the (021) and (002) diffraction planes of FeOOH can also be seen in the SAED image (Figure S14, Supporting Information). The elemental mapping (Figure S15, Supporting Information) also demonstrates the existence of Ni, Fe, and O elements alongside their homogenous distribution throughout the whole heterostructure, similar to the initial sample. These results certify that the morphology of the OER‐tested NiFe‐LDH/FeOOH is identical to that of the initial sample, exhibiting superior catalytic stability.

The XRD pattern in Figure S16 (Supporting Information) displays the comparison of NiFe‐LDH/FeOOH before and after the OER test, the similar diffraction peaks confirm the structural stability of NiFe‐LDH/FeOOH. Figure 7e shows the Raman spectrum of NiFe‐LDH/FeOOH before and after OER, and a blue shift is observed, namely Raman peak shifts to a higher wavenumber in the NiFe‐LDH/FeOOH after the stability test, indicating that the bond energy of M─O bonds is enhanced. Moreover, the relative intensity reduction of Fe^3+^─O peak corresponding to NiFe‐LDH after the OER stability test suggests a decrease in the quantity of Fe^3+^─O bonds due to the involvement of Fe─O synergistic sites in NiFe‐LDH during the OER reaction, in agreement with the above results. Furthermore, the XPS spectra of OER‐tested NiFe‐LDH/FeOOH illustrate the changes in valence states after the stability test (Figure 7f–h). Interestingly, Fe 2p and O 1s after the OER test exhibit a positive shift compared to those in the initial NiFe‐LDH/FeOOH, suggesting that Fe 2p and O 1s lose electrons and their valence states increase, while the binding energy of Ni 2p shifts to a lower level owing to the electrons gained and its decrease of valence state. The changes can arise from the fact that O‐lattice removes oxygen atoms to form oxygen defects accompanied by the positive shift of O‐adsorbed and O‐defective, consistent with previous studies.^[^ ^37^, ^46^ ^]^ The variation of oxygen vacancy concentration before and after the OER reaction can be demonstrated by EPR tests (Figure 5f). In addition, Fe sites with higher oxidation valence states are more beneficial for OER activity, since Fe doping can stabilize highly active sites at low potentials to enhance catalytic activity and promote electrochemical water oxidation.^[^ ^47^ ^]^

### 2.4 Mechanism Study of the OER Activity of NiFe‐LDH/FeOOH

**Figure 8:**
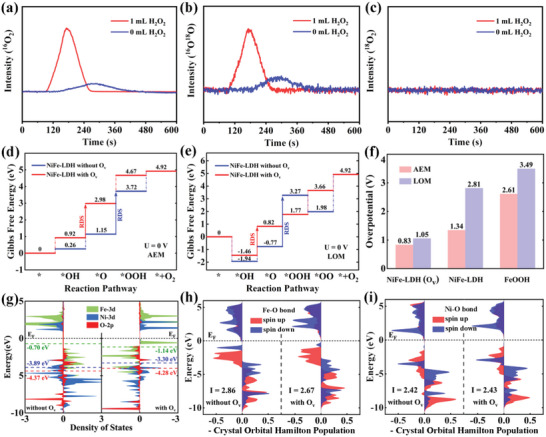
Investigation of OER cycling for NiFe‐LDH/FeOOH with and without oxygen defects. a–c) The results of differential electrochemical mass spectrometry (DEMS) measurements for NiFe‐LDH/FeOOH synthesized with 0 and 1 mL H_2_O_2_ indicate the variation of different gaseous product content during OER, where the lattice oxygen is labeled by the ^18^O isotope. d,e) The Gibbs free energy diagrams of AEM and LOM pathways on NiFe‐LDH, where the arrows show the rate‐determining steps (RDS). f) Overpotentials of NiFe‐LDH with and without O_V_ and FeOOH for AEM and LOM. g) Projected density of states of NiFe‐LDH. The numbers show the centers of corresponding bands. h,i) ‐Crystal Orbital Hamilton Population (‐COHP) of Fe─O and Ni─O bond. “*I*” refers to the integrated ‐COHP under the Fermi level.

To elucidate the reaction pathway and mechanisms of superior catalytic performance of NiFe‐LDH/FeOOH heterostructures with oxygen defects, isotope labeling experiments, DEMS measurements, and density functional theory (DFT) calculations are performed for NiFe‐LDH/FeOOH with different oxygen vacancy contents. Typically, oxygen generation via OER with the involvement of lattice oxygen is confirmed by ^18^O isotope labeling experiments. DEMS measurements (**Figure** **8**a–c) of ^18^O‐labeled NiFe‐LDH/FeOOH show signals of m/z = 32 and m/z = 34, while no signal corresponding to m/z = 36 can be detected, indicating the generation of ^16^O_2_ and ^16^O^18^O instead of ^18^O_2_ during the OER process. The results demonstrate that the OER over NiFe‐LDH/FeOOH follows the synergy mechanism of AEM and LOM mechanisms. It is remarkable that the reaction pathway of LOM in this work obeys the single lattice oxygen mechanism (sLOM) as shown in Figure 1c, with oxygen production from the combination of lattice oxygen and adsorbed oxygen, rather than from two lattice oxygen in double lattice oxygen mechanism (dLOM) as reported in previous works (Figure 1d).^[^ ^22^, ^27^ ^]^ The sLOM favors the OER process since the O─O bond formation from two lattice oxygen in dLOM is energetically unfavorable, which is undesirable for the catalytic reaction to proceed.^[^ ^22^, ^32^ ^]^ Besides, the variation in the intensity of O_2_ produced over different catalysts can be demonstrated by the real‐time gas product content. Compared with the non‐H_2_O_2_ sample, NiFe‐LDH/FeOOH synthesized with 1 mL H_2_O_2_ has significantly higher ^16^O_2_ and ^16^O^18^O contents in the products, which implies that the oxygen defects contribute to both AEM and sLOM mechanisms. The synergy of AEM and sLOM can maximize the use of active sites on the catalyst surface, including metal sites and oxygen sites, and thus enhance the catalytic performance. Note that the sLOM was recently also detected in different systems, although it was not distinguished from the dLOM.^[^ ^32^, ^44^, ^48^ ^]^ In addition, the sLOM in previous works was triggered by regulating the metal cations and was usually accompanied by the dLOM, in other words, showing the mixed mechanism of sLOM and dLOM. Essentially different from the methods that will induce the mixed mechanism, the oxygen defect engineering presented in this work only triggers the sLOM, and is a more facile and effective strategy to promote LOM.

In addition to the DEMS measurements, DFT calculations are performed to determine the OER mechanism on NiFe‐LDH/FeOOH, where both AEM and sLOM routes are taken into account (Figure 1a,c). For the AEM route, four proton‐electron concerted processes are taken into account, including the fundamental processes of *OH formation, *OH deprotonation, *OOH formation (the asterisk denotes the adsorption site), and O_2_ release.^[^ ^49^ ^]^ Regarding the sLOM route, the lattice oxygen also participates in the OER cycle as part of the intermediate, and because the lattice oxygen stably bridges between metal atoms, *OOH deprotonation can occur to break the linear scaling relation (LSR) for AEM.^[^ ^32^ ^]^ For an in‐depth understanding of the role of AEM and sLOM routes in OER, comprehensive DFT theoretical calculations of reaction free‐energy landscapes are performed. The Gibbs free energies (Δ*G*) of Fe and lattice oxygen sites in FeOOH are first investigated to explore the real active sites (Figure S17, Supporting Information). From the ΔG landscape, the deprotonation of *OH serves as the rate‐determining step (RDS) in both AEM and sLOM routes, with a higher overpotential of 2.61 and 3.49 V, respectively, noting that the formation of *OO cannot reduce the barrier of the deprotonation of *OH in sLOM. The large thermodynamic overpotentials indicate that the FeOOH has poor activity. Next, the ΔG calculations are performed in NiFe‐LDH with and without oxygen vacancy (O_V_) because the oxygen defects are found in our samples, and the optimized geometric structures and adsorption intermediates are depicted in Figure S18‐S19 (Supporting Information). In the AEM route (Figure 8d), the Fe sites are found to be the active sites, and the formation of *OOH is the RDS for NiFe‐LDH, with an overpotential of 1.34 V. After the introduction of O_V_, all intermediates (*OH, *O, and *OOH) hold lower free energy than pure NiFe‐LDH, indicating the adsorption between the Fe sites and intermediates are weakened. Unexpectedly, the RDS is the transition from the formation of *OOH to the deprotonation of *OH with a lower overpotential of 0.83 V, demonstrating that the O_V_ can improve the catalytic activity of Fe sites. In the LOM route (Figure 8e), the thermodynamic overpotential of NiFe‐LDH is high (≈2.81 V), indicating that the activity of lattice oxygen is poor due to the strong adsorption between Fe or Ni site and intermediates. Interestingly, when introducing O_V_, the thermodynamic overpotential significantly reduced to 1.05 V with the change of the RDS from *OOH formation to *OH deprotonation, indicating that the O_V_ can effectively activate lattice oxygen in NiFe‐LDH. Based on the results given above (Figure 8f), when there is no oxygen vacancy, the overpotential of the sLOM is twice as high as that of the AEM, and it can be approximated that at this time all OER reactions are carried out via the AEM route. However, when there are oxygen vacancies, not only the overpotentials of sLOM and AEM are both decreased, but also the ratio of their overpotentials is reduced to 1.26. It is worth noting that the close overpotentials enable AEM and sLOM mechanisms to proceed simultaneously and promote the synergy between AEM and sLOM routes of OER. Consequently, the catalytic activity of NiFe‐LDH/FeOOH can be greatly improved, which is consistent with the results of DEMS measurements.

To give further insight into the influence of O_V_ on improved OER performance, the projected density of states (PDOS) with band centers of Fe, Ni, and O sites at NiFe‐LDH with and without O_V_ are investigated and displayed in Figure 8g. For NiFe‐LDH both with and without O_V_, PDOS of Fe is closer to the Fermi level than that of Ni, thus providing stronger adsorption stability. When the O_V_ is introduced in NiFe‐LDH, the Fe‐3d band center shifts down, which could effectively reduce the over‐strong adsorption ability of Fe sites to the intermediates or lattice oxygen. Meanwhile, O_V_ also causes the 2*p* band of lattice oxygen to rise, indicating that the lattice oxygen is activated, i.e., the lattice oxygen obtains electrons to form O_2_ more easily. Moreover, the lattice oxygen activity can also be estimated by the metal─oxygen bond strength, which can be evaluated by calculating the crystal orbital Hamilton populations (COHP) in Figure 8h,i. The negative and positive values of ‐COHP correspond to the anti‐bonding and bonding states, respectively. To quantify the metal─oxygen bond strength, the integral of ‐COHP (I) up to the Fermi level of Fe─O and Ni─O bonds are determined to be 2.86 (2.67) and 2.42 (2.43) for NiFe‐LDH without (with) O_V_, respectively. For the Fe─O bond, the smaller value of I for NiFe‐LDH with O_V_ indicates that introducing O_V_ results in more electrons filled into the anti‐bonding orbitals, leading to the weaker metal─oxygen bond, which is beneficial to activate lattice oxygen. For the Ni─O bond, there is nearly no difference in the value of I in NiFe‐LDH with and without O_V_ due to the lower *d*‐band center of Ni. Therefore, introducing O_V_ will shift the Fe‐3*d* band center down, which not only reduces the adsorption strength between Fe and the intermediate to improve catalytic activity for AEM but also weakens the Fe─O bond to promote activation of the lattice oxygen for sLOM. This further discloses that the oxygen vacancy can promote the synergy between AEM and sLOM routes of OER in NiFe‐LDH/FeOOH heterostructures.

## 3 Conclusion

In summary, a 3D open architecture formed of vertically 2D hexagonal NiFe‐LDH/FeOOH nanosheets with oxygen defects is generated on NF, using a one‐step hydrothermal technique based on an in situ competitive growth strategy of bimetallic ions and OH^−^ ions. More importantly, a combination of controlled experiments and theoretical calculations reveal that the ratio of NiFe‐LDH and FeOOH, metal valence, the content of oxygen defects, and the electrocatalytic activity of NiFe‐LDH/FeOOH could be easily controlled by *only* changing the H_2_O_2_ addition. Specifically, NiFe‐LDH/FeOOH prepared with appropriate H_2_O_2_ addition has a high ratio of oxygen defects, which can tune the metal─oxygen covalency, promote the Fe─O sites synergy, and effectively enhance the catalytic performance. Furthermore, DEMS measurements and DFT calculations account for the enhanced catalytic performance, suggesting the contribution of oxygen defects to the synergy between AEM and sLOM mechanisms. Impressively, the optimized NiFe‐LDH/FeOOH catalyst requires an overpotential of only 177 mV to reach the current density at 20 mA cm^−2^ and can reach the industrial‐level current density of 1 A cm^−2^ in 1.0 M KOH electrolyte. The growth mechanism of NiFe‐LDH/FeOOH can be generalized in the formation of TM‐based catalysts, providing a new strategy through constructing oxygen defects to obtain low‐cost and highly efficient OER electrocatalysts.

## Conflict of Interest

The authors declare no conflict of interest.

## Supporting information

Supporting Information

## Data Availability

The data that support the findings of this study are available from the corresponding author upon reasonable request.

## References

[1] X. H. Xie , L. Du , L. T. Yon , S. Y. Park , Y. Qiu , J. Sokolowski , W. Wang , Y. Y. Shao , Adv. Funct. Mater. 2022, 32, 2110036.

[2] J. Zhang , W. J. Jiang , S. Niu , H. T. Zhang , J. Liu , H. Y. Li , G. F. Huang , L. Jiang , W. Q. Huang , J. S. Hu , W. P. Hu , Adv. Mater. 2020, 32, 1906015. 10.1002/adma.201906015 32027058

[3] D. Philo , S. Q. Luo , C. He , Q. Wang , F. Ichihara , L. L. Jia , M. Oshikiri , H. Pang , Y. Wang , S. J. Li , G. L. Yang , X. H. Ren , H. W. Lin , J. H. Ye , Adv. Funct. Mater. 2022, 32, 2206811.

[4] B. Zhang , X. L. Zheng , O. Voznyy , R. Comin , M. Bajdich , M. Garcia‐Melchor , L. L. Han , J. X. Xu , M. Liu , L. R. Zheng , F. P. G. de Arquer , C. T. Dinh , F. J. Fan , M. J. Yuan , E. Yassitepe , N. Chen , T. Regier , P. F. Liu , Y. H. Li , P. De Luna , A. Janmohamed , H. L. L. Xin , H. G. Yang , A. Vojvodic , E. H. Sargent , Science 2016, 352, 333. 27013427 10.1126/science.aaf1525

[5] F. Dionigi , Z. H. Zeng , I. Sinev , T. Merzdorf , S. Deshpande , M. B. Lopez , S. Kunze , I. Zegkinoglou , H. Sarodnik , D. X. Fan , A. Bergmann , J. Drnec , J. F. de Araujo , M. Gliech , D. Teschner , J. Zhu , W. X. Li , J. Greeley , B. Roldan Cuenya , P. Strasser , Nat. Commun. 2020, 11, 2522. 32433529 10.1038/s41467-020-16237-1 PMC7239861

[6] X. L. Yi , L. Z. Song , S. X. Ouyang , N. Wang , H. Y. Chen , J. B. Wang , J. Lv , J. H. Ye , Small 2021, 17, 2102222. 10.1002/smll.202102222 34411433

[7] L. G. Zhang , W. Tang , C. Dong , D. X. Zhou , X. Q. Xing , W. J. Dong , Y. H. Ding , G. Wang , M. Y. Wu , Appl. Catal. B‐Environ. 2022, 302, 120833.

[8] J. Y. Xie , C. Li , J. Niu , S. H. Zhang , X. M. Ou , P. Z. Feng , H. Garcia , Mater. Lett. 2021, 290, 129517.

[9] G. Y. Shi , T. Tano , D. A. Tryk , M. Yamaguchi , A. Iiyama , M. Uchida , K. Iida , C. Arata , S. Watanabe , K. Kakinuma , ACS Catal. 2022, 12, 14209. 10.1021/acsomega.3c00322 PMC10099113 37065081

[10] B. Li , Z. Tian , L. Li , Y. H. Wang , Y. Si , H. Wan , J. H. Shi , G. F. Huang , W. Y. Hu , A. L. Pan , W. Q. Huang , ACS Nano 2023, 17, 3465. 36763083 10.1021/acsnano.2c09659

[11] C. G. Kuai , Y. Zhang , D. Y. Wu , D. Sokaras , L. Q. Mu , S. Spence , D. Nordlund , F. Lin , X. W. Du , ACS Catal. 2019, 9, 6027.

[12] J. Y. C. Chen , L. N. Dang , H. F. Liang , W. L. Bi , J. B. Gerken , S. Jin , E. E. Alp , S. S. Stahl , J. Am. Chem. Soc. 2015, 137, 15090. 26601790 10.1021/jacs.5b10699

[13] H. S. Ahn , A. J. Bard , J. Am. Chem. Soc. 2016, 138, 313. 26645678 10.1021/jacs.5b10977

[14] A. Grimaud , A. Demortiere , M. Saubanere , W. Dachraoui , M. Duchamp , M. L. Doublet , J. M. Tarascon , Nat. Energy 2017, 2, 16189.

[15] J. Suntivich , K. J. May , H. A. Gasteiger , J. B. Goodenough , Y. Shao‐Horn , Science 2011, 334, 1383. 22033519 10.1126/science.1212858

[16] J. Hwang , R. R. Rao , L. Giordano , Y. Katayama , Y. Yu , Y. Shao‐Horn , Science 2017, 358, 751. 29123062 10.1126/science.aam7092

[17] T. H. Jeon , S. Han , B. Kim , C. Park , W. Kim , H. Park , W. Y. Choi , ACS Energy Lett. 2022, 7, 59.

[18] G. G. Liu , P. Li , G. X. Zhao , X. Wang , J. T. Kong , H. M. Liu , H. B. Zhang , K. Chang , X. G. Meng , T. Kako , J. H. Ye , J. Am. Chem. Soc. 2016, 138, 9128. 27380539 10.1021/jacs.6b05190

[19] Y. L. Zhu , H. A. Tahini , Z. W. Hu , Z. G. Chen , W. Zhou , A. C. Komarek , Q. Lin , H. J. Lin , C. T. Chen , Y. J. Zhong , M. T. Fernandez‐Diaz , S. C. Smith , H. T. Wang , M. L. Liu , Z. P. Shao , Adv. Mater. 2020, 32, 1905025. 10.1002/adma.201905025 31713899

[20] N. Zhang , Y. Chai , Energy Environ. Sci. 2021, 14, 4647.

[21] D. A. Kuznetsov , M. A. Naeem , P. V. Kumar , P. M. Abdala , A. Fedorov , C. R. Muller , J. Am. Chem. Soc. 2020, 142, 7883. 32216262 10.1021/jacs.0c01135

[22] A. Grimaud , O. Diaz‐Morales , B. H. Han , W. T. Hong , Y. L. Lee , L. Giordano , K. A. Stoerzinger , M. T. M. Koper , Y. Shao‐Horn , Nat. Chem. 2017, 9, 457. 28430191 10.1038/nchem.2695

[23] A. Grimaud , W. T. Hong , Y. Shao‐Horn , J. M. Tarascon , Nat. Mater. 2016, 15, 121. 26796721 10.1038/nmat4551

[24] Z. F. Huang , J. J. Song , Y. H. Du , S. B. Xi , S. Dou , J. M. V. Nsanzimana , C. Wang , Z. C. J. Xu , X. Wang , Nat. Energy 2019, 4, 329.

[25] M. Kim , J. Park , M. Kang , J. Y. Kim , S. W. Lee , Acs Central Sci 2020, 6, 880. 10.1021/acscentsci.0c00479 PMC7318066 32607435

[26] J. S. Yoo , X. Rong , Y. S. Liu , A. M. Kolpak , ACS Catal. 2018, 8, 4628.

[27] X. P. Wang , S. B. Xi , P. R. Huang , Y. H. Du , H. Y. Zhong , Q. Wang , A. Borgna , Y. W. Zhang , Z. B. Wang , H. Wang , Z. G. Yu , W. S. V. Lee , J. M. Xue , Nature 2022, 611, 702. 36289339 10.1038/s41586-022-05296-7

[28] J. J. Song , C. Wei , Z. F. Huang , C. T. Liu , L. Zeng , X. Wang , Z. C. J. Xu , Chem. Soc. Rev. 2020, 49, 2196. 32133479 10.1039/c9cs00607a

[29] S. Anantharaj , S. Kundu , S. Noda , Nano Energy 2021, 80, 105514.

[30] W. Zhao , H. Xu , H. Luan , N. Chen , P. Gong , K. Yao , Y. Shen , Y. Shao , Adv. Energy Mater. 2021, 12, 2102372.

[31] J. He , X. Zhou , P. Xu , J. M. Sun , Nano Energy 2021, 80, 105540.

[32] Z. Y. He , J. Zhang , Z. H. Gong , H. Lei , D. Zhou , N. Zhang , W. J. Mai , S. J. Zhao , Y. Chen , Nat. Commun. 2022, 13, 2191. 35449165 10.1038/s41467-022-29875-4 PMC9023528

[33] D. Liu , H. Q. Ai , J. L. Li , M. L. Fang , M. P. Chen , D. Liu , X. Y. Du , P. F. Zhou , F. F. Li , K. H. Lo , Y. X. Tang , S. Chen , L. Wang , G. C. Xing , H. Pan , Adv. Energy Mater. 2020, 10, 2002464.

[34] Y. Y. Zhai , X. R. Ren , Y. Sun , D. Li , B. L. Wang , S. Liu , Appl. Catal. B‐Environ. 2023, 323, 122091.

[35] T. Wang , X. Li , Y. Pang , X. Gao , Z. Kou , J. Tang , J. Wang , Chem. Eng. J. 2021, 425, 131491.

[36] K. Jiang , W. J. Liu , W. Lai , M. L. Wang , Q. Li , Z. L. Wang , J. J. Yuan , Y. L. Deng , J. Bao , H. B. Ji , Inorg. Chem. 2021, 60, 17371. 34705457 10.1021/acs.inorgchem.1c02903

[37] J. J. Lv , L. M. Wang , R. S. Li , K. Y. Zhang , D. F. Zhao , Y. Q. Li , X. J. Li , X. B. Huang , G. Wang , ACS Catal. 2021, 11, 14338.

[38] D. S. Hall , D. J. Lockwood , S. Poirier , C. Bock , B. R. MacDougall , J. Phys. Chem. A 2012, 116, 6771. 22642317 10.1021/jp303546r

[39] J. D. Chen , F. Zheng , S. J. Zhang , A. Fisher , Y. Zhou , Z. Y. Wang , Y. Y. Li , B. B. Xu , J. T. Li , S. G. Sun , ACS Catal. 2018, 8, 11342.

[40] J. L. Li , L. B. Yao , D. Z. Wu , J. King , S. S. C. Chuang , B. Liu , Z. M. Peng , Appl. Catal. B‐Environ. 2022, 316, 121657, 121657.

[41] Y. Wang , X. Li , M. Zhang , J. Zhang , Z. Chen , X. Zheng , Z. Tian , N. Zhao , X. Han , K. Zaghib , Y. Wang , Y. Deng , W. Hu , Adv. Mater. 2022, 34, 2107053. 10.1002/adma.202107053 35080286

[42] Z. H. Yan , H. M. Sun , X. Chen , H. H. Liu , Y. R. Zhao , H. X. Li , W. Xie , F. Y. Cheng , J. Chen , Nat. Commun. 2018, 9, 2373. 29915288 10.1038/s41467-018-04788-3 PMC6006371

[43] B. C. Qiu , C. Wang , N. Zhang , L. J. Cai , Y. J. Xiong , Y. Chai , ACS Catal. 2019, 9, 6484.

[44] N. Zhang , X. Feng , D. Rao , X. Deng , L. Cai , B. Qiu , R. Long , Y. Xiong , Y. Lu , Y. Chai , Nat. Commun. 2020, 11, 4066. 32792524 10.1038/s41467-020-17934-7 PMC7426847

[45] J. T. Mefford , X. Rong , A. M. Abakumov , W. G. Hardin , S. Dai , A. M. Kolpak , K. P. Johnston , K. J. Stevenson , Nat. Commun. 2016, 7, 11053. 27006166 10.1038/ncomms11053 PMC4814573

[46] H. Liu , X. N. Li , C. L. Peng , L. Y. Zhu , Y. X. Zhang , H. R. Cheng , J. M. Cui , Q. M. Wu , Y. Y. Zhang , Z. Z. Chen , W. Zou , G. Wen , H. L. Huang , J. L. Wang , B. J. Ye , Z. P. Fu , Y. L. Lu , J. Mater. Chem. A 2020, 8, 13150.

[47] Y. Yang , L. N. Dang , M. J. Shearer , H. Y. Sheng , W. J. Li , J. Chen , P. Xiao , Y. H. Zhang , R. J. Hamers , S. Jin , Adv. Energy Mater. 2018, 8, 1703189.

[48] H. J. Zhang , Y. X. Gao , H. Y. Xu , D. Q. Guan , Z. W. Hu , C. Jing , W. Zhou , Z. P. Shao , Adv. Funct. Mater. 2022, 32, 2207618.

[49] X. Wang , X. Wan , X. Qin , ACS Catal. 2022, 12, 9437.

