# Supplementary material for: Oxygen Defect Engineering Promotes Synergy Between Adsorbate Evolution and Single Lattice Oxygen Mechanisms of OER in Transition Metal‐Based (oxy)Hydroxide
Source: Adv Sci (Weinh). 2023 Oct 9;10(32):2303321. doi: 10.1002/advs.202303321 (PMC10646268; doi:10.1002/advs.202303321)
Supplement: Supplementary file 1 — Supporting Information [file ADVS-10-2303321-s001.pdf]

ADVANCED SCIENCE
Open Access

Supporting Information

for *Adv. Sci.*, DOI 10.1002/advs.202303321

Oxygen Defect Engineering Promotes Synergy Between Adsorbate Evolution and Single Lattice Oxygen Mechanisms of OER in Transition Metal-Based (oxy)Hydroxide

Yu-Han Wang, Lei Li, Jinghui Shi, Meng-Yuan Xie, Jianhang Nie, Gui-Fang Huang, Bo Li*, Wangyu Hu, Anlian Pan and Wei-Qing Huang**

Supporting Information

Oxygen Defect Engineering Promotes Synergy Between Adsorbate Evolution and Single Lattice Oxygen Mechanisms of OER in Transition Metal-based (oxy)Hydroxide

Yu-Han Wang, Lei Li, Jinghui Shi, Meng-Yuan Xie, Jianhang Nie, Gui-Fang Huang, Bo Li,* Wangyu Hu, Anlian Pan, and Wei-Qing Huang**

Y. H. Wang, Dr. L. Li, Dr. J. H. Shi, Dr. M.Y. Xie, Dr. J. H. Nie, Prof. G. F. Huang, Dr. B. Li, and Prof. W. Q. Huang
Department of Applied Physics, School of Physics and Electronics
Hunan University
Changsha, 410082, P. R. China

*E-mail: gfhuang@hnu.edu.cn
*E-mail: boli1993@hnu.edu.cn
*E-mail: wqhuang@hnu.edu.cn

Prof. W. Y. Hu, and Prof. A. L. Pan
School of Materials Science and Engineering
Hunan University
Changsha, 410082, P. R. China

Experimental Section

***Chemicals and Reagents*:** Ni foam (1.7 mm in thickness, Taiyuan Lizhiyuan Science and Technology Co., P.R. China), hydrochloric acid (Sinopharm Chemical Reagent Co., P. R. China), acetone (Sinopharm Chemical Reagent Co., P. R. China), ethanol (Tianjin Zhiyuan Chemical Reagent Co., P. R. China), urea (Shanghai Aibi Chemical Reagent Co., P. R. China), $Fe(NO_3)_3{\cdot}9H_2O$ (Sinopharm Chemical Reagent Co., P. R. China), H_2O_2 (Tianjin Zhiyuan Chemical Reagent Co., P. R. China). The chemicals were used as received without further purification.

***Pretreatment of NF substrates*:** A piece of NF (2cm*3cm*1.7mm) was sonicated with concentrated hydrochloric acid, acetone, ethanol, and deionized water for 10 minutes, respectively. After sonication, the NF was rinsed repeatedly with copious deionized (DI) water.

***Synthesis of NiFe-LDH/FeOOH*:** 180 mg urea and 1mmol $Fe(NO_3)_3{\cdot}9H_2O$ were dissolved in 30 mL DI water. Then 1 mL H_2O_2 (30 wt%) was added to the solution under continuous stirring. After that, the pre-cleaned NF substrate was immersed into the above solution and hydrothermally treated at 120 °C for 12 h. After cooling to room temperature, the sample was taken out and repeatedly washed with ethanol and DI water. The cleaned sample was then dried at 60 °C for 4 hours.

***Synthesis of Ni(OH)₂*:** For comparison, the sample was prepared by a method similar to that for the NiFe-LDH/FeOOH in the absence of $Fe(NO_3)_3{\cdot}9H_2O$.

***Structural Characterization*:** SEM and EDS elemental mapping measurements were performed on a field emission scanning electron microscope (SEM, Hitachi S-4800, equipped with energy dispersive X-ray detector). A transmission electron microscope (TEM) and a high-resolution TEM (HRTEM) were used (TEM, JEM-3010, Japan) to determine the morphology and microstructure of samples. X-ray diffractometry (XRD) measurements were carried out on a Siemens D-5000 diffractometer using Cu Kα radiation (λ=0.154 nm). Raman spectroscopy was conducted on a Renishaw inVia Raman microscope. A PHI Quantera X-ray photoelectron spectrometer with an Al Kα source (300W) was used to perform X-ray photoelectron spectroscopy (XPS).

***Electrochemical Characterization*:** All OER tests were carried out on a CHI760E workstation in a three-electrode system with the as-synthesized catalysts, Pt sheet, and Ag/AgCl electrode in saturated KCl solution serving as the working, counter, and reference electrodes, respectively. 1.0 M KOH with saturated O_2 was applied as the

electrolyte. And both sides of every working electrode are estimated. The linear sweep voltammetry (LSV) polarization curves were measured at a rate of 2 mV s^{-1}. The staircase cyclic voltammetry (SCV) curves were tested with the potential increment of 4 mV. All polarization curves were corrected by 85% iR compensation for ohmic losses arising from active materials, substrate, and solution resistances. The electrochemical impedance spectroscopy (EIS) measurements are carried out in the frequency range from 1 MHz to 0.05 Hz. The chronopotentiometry was performed with a constant current density of 20, 100, and 1000 mA cm^{-2} in an O_2-saturated 1.0 M KOH solution. The Tafel slope is calculated according to the equation: $\eta = b \times \log |j| + a$, where η is the overpotential (V), j is the current density (mA cm^{-2}), and b is the Tafel slope (mV dec^{-1}). The C_{dl} was calculated by taking CV measurements at varied scan rates of 20, 40, 60, 80, 100, 120, 140, 160, 180, and 200 mV/s. The value of C_{dl} was the linear slope obtained from plotting $\Delta j = j_a - j_c$ against the scan rates (j_a and j_c are anode and cathode current density, respectively). All the potentials were calibrated to the reversible hydrogen electrode (vs. RHE), and the corresponding equation is $E_{RHE} = E_{Ag/AgCl} +$ 1.022 V.

^{18}O-labeling experiment: NiFe-LDH/FeOOH with 0 and 1 mL H_2O_2 were labeled with ^{18}O isotopes by potentiostatic reaction for 30 min in KOH solution with $H_2{}^{18}O$. The ^{18}O-labeled catalysts were then rinsed several times with $H_2{}^{16}O$ to remove the remaining $H_2{}^{18}O$.

DEMS measurements: DEMS measurements were carried out by a QAS 100 device (Linglu Instruments, Shanghai). The NiFe-LDH/FeOOH with ^{18}O-labeling, Pt sheet, and Ag/AgCl electrode in saturated KCl solution were used as working, counter, and reference electrode, respectively. CV measurement was performed in KOH solution with $H_2{}^{16}O$ with a scan rate of 5 mV/s. Meanwhile, the gas products of different molecular weights were monitored by mass spectroscopy in real time.

Theoretical Calculations

The Spin-polarized DFT calculations were performed on Vienna ab initio Simulation package (VASP)[1,2] with projector augmented wave (PAW)[3,4] pseudopotential. The exchange-correlation interaction was described by Perdew-Burke-Ernzerhof (PBE) functional within the generalized gradient approximation (GGA).[5] The kinetic energy cut-off was set to 500 eV, and the dispersion corrections in Grimme's scheme (DFT-

D3) was utilized to treat the long-range van der Waals interactions.[6,7] The Brillouin zone was sampled by Gamma-centered *k*-point with 1 × 1 × 1,[8] and the convergence criterion of force and energy were set to 0.01 eV $Å^{-1}$ and 10^{-5} eV, respectively. To better describe the localized 3*d* orbital, the effective U value of Ni and Fe atoms were set to 2.56 and 5.2 eV, respectively.[9]

The Gibbs free energy are calculated to investigate the OER performances. For the AEM pathway in an alkaline electrolyte, the four-electron reactions can be described as:

$$* + OH^- \rightarrow *OH + e^-,$$

$$*OH + OH^- \rightarrow *O + H_2O + e^-,$$

$$*O + OH^- \rightarrow *OOH + e^-,$$

$$*OOH + OH^- \rightarrow * + O_2 + H_2O + e^-,$$

where "*" represents the adsorption sites, which are generally the exposed metal sites. The Gibbs free energy of each step can be calculated as:

$$\Delta G_{*OH} = G_{*OH} - G_{*} - G_{H2O} + 1/2G_{H2},$$

$$\Delta G_{*O} = G_{*O} - G_{*} - G_{H2O} + G_{H2},$$

$$\Delta G_{*OOH} = G_{*OOH} - G_{*} - 2G_{H2O} + 3/2G_{H2},$$

$$\Delta G_{*+O2} = 2G_{H2} + G_{O2} - 2G_{H2O},$$

where G_{O2} is the Gibbs free energies of O_2 which can be calculated as G_{O2} = 4.92 eV – $2G_{H2}$ + $2G_{H2O}$ and the G_{*}, G_{*OH}, G_{*O}, G_{*OOH}, G_{*+O2}, G_{H2O} and G_{H2} are the Gibbs free energies of *, *OH, *O, *OOH, *+O_2, H_2O and H_2 molecules, respectively, which can be calculated by G = E + ZPE – TS, where E is the calculated total energy from DFT calculation, and ZPE, TS and G are the zero-point energy correction, entropy, and free energy terms, respectively. The step with the greatest increasing energy of OER is defined as the rate-determining step (RDS), which decides the energy barrier for the whole reaction. To evaluate the catalytic activity, the OER overpotential (η_{OER}) in the normal hydrogen electrode (NHE) is defined as: η_{OER} = max {ΔG_{*OH}, $\Delta G_{*O} - \Delta G_{*OH}$, $\Delta G_{*OOH} - \Delta G_{*O}$, $\Delta G_{*+O2} - \Delta G_{*OOH}$}/e – 1.23.

The LOM pathway includes five steps:

$$*O_lH + OH^- \rightarrow *O_l + H_2O + e^-,$$

$$*O_l + OH^- \rightarrow *O_lOH + e^-,$$

$$*O_lOH + OH^- \rightarrow *O_lO + H_2O + e^-,$$

$$*O_lO \rightarrow * + O_2,$$

$$* + OH^- \rightarrow *O_lH + e^-,$$

where "*" stands for the vacancy sites, and "O_l" for the lattice oxygen atoms. The Gibbs free energy of each step can be calculated as:

$$\Delta G_{*OH} = G_{*OH} - G_{*} - G_{H2O} + 1/2G_{H2},$$

$$\Delta G_{*O} = G_{*O} - G_{*} - G_{H2O} + G_{H2},$$

$$\Delta G_{*OOH} = G_{*OOH} - G_{*} - 2G_{H2O} + 3/2G_{H2},$$

$$\Delta G_{*OO} = G_{*OO} - G_{*} - 2G_{H2O} + 2G_{H2},$$

$$\Delta G_{*+O2} = 2G_{H2} + G_{O2} - 2G_{H2O},$$

where the G_{*}, G_{*OH}, G_{*O}, G_{*OOH}, G_{*OO}, G_{*+O2}, G_{H2O}, G_{O2} and G_{H2} are the Gibbs free energies of *, $*O_lH$, $*O_l$, $*O_lOH$, $*O_lO$, $*+O_2$, H_2O, O_2 and H_2 molecules, respectively. For the catalytic activity, the OER overpotential (η_{OER}) in the normal hydrogen electrode (NHE) is defined as: η_{OER} = max $\{\Delta G_{*OH}, \Delta G_{*O}–\Delta G_{*OH}, \Delta G_{*OOH}–\Delta G_{*O}, \Delta G_{*OO}–\Delta G_{*OOH}, \Delta G_{*+O2}–\Delta G_{*OOH}\}/e - 1.23$.

Growth Mechanism of NiFe-LDH/FeOOH Heterostructure

Under hydrothermal conditions, urea is slowly hydrolyzed to produce NH_4^+ and CO_3^{2-} (Equation 1), which would further be hydrolyzed to generate H^+ (Equation 2) and OH^- (Equation 3) ions, respectively. The produced OH^- ions will coprecipitate with Fe^{3+} ions in the solution to form FeOOH (Equation 4); meanwhile, the produced H^+ ions will etch NF to generate Ni^{2+} ions (Equation 5). Since the urea hydrolysis is relatively weak, only small amount of Ni^{2+} ions are generated from the NF etching and will coprecipitate with Fe^{3+} ions to form NiFe-LDH lobulate nanosheets (Equation 6). As H_2O_2 is added into the reaction solution, the Fenton-like and Fenton reactions take place (Equation 7, 8), resulting in large quantities of hydroxyl radicals (*OH) and OH^- ions. The strongly oxidative *OH would accelerate the etching of NF to produce more Ni^{2+} ions and enhance the localized alkaline environment near the NF surface (Equation 9). As a result, more Ni^{2+} and Fe^{3+} ions will coprecipitate with OH^- ions to form abundant NiFe-LDH (Equation 6), besides Fe^{3+} ions bond with OH^- ions forming FeOOH (Equation 4). This competing interaction between metal ions (Ni^{2+} and Fe^{3+}) with OH^- ions will lead to the formation of NiFe-LDH/FeOOH heterostructure accompanying with oxygen defects.

$$CO(NH_2)_2 + 2H_2O \rightarrow 2NH_4^+ + CO_3^{2-} \quad (1)$$

$$NH_4^+ + H_2O \rightarrow NH_3{\cdot}H_2O + H^+ \quad (2)$$

$$CO_3^{2-} + H_2O \rightarrow HCO_3^- + OH^- \tag{3}$$

$$Fe^{3+} + 3OH^- \rightarrow FeOOH + H_2O \tag{4}$$

$$Ni + 2H^+ \rightarrow Ni^{2+} + H_2 \tag{5}$$

$$Ni^{2+} + Fe^{3+} + CO_3^{2-} + OH^- \rightarrow NiFe\text{-}LDH \tag{6}$$

$$Fe^{3+} + H_2O_2 \rightarrow Fe^{2+} + {}^*HO_2 + H^+ \tag{7}$$

$$Fe^{2+} + H_2O_2 \rightarrow Fe^{3+} + {}^*OH + OH^- \tag{8}$$

$$Ni + 2{}^*OH \rightarrow Ni^{2+} + 2OH^- \tag{9}$$

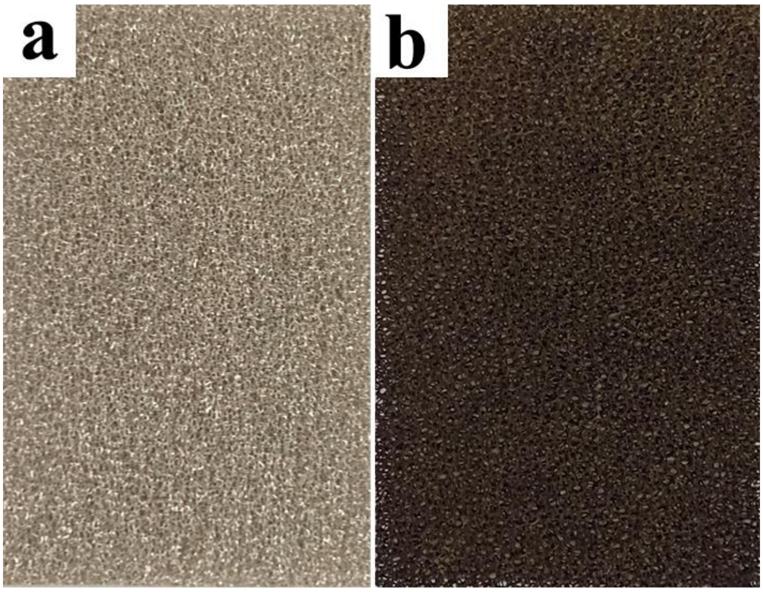

Figure S1. Optical images of a) Ni foam, and b) NiFe-LDH/FeOOH.

Using NF as the nickel source and conductive substrate, the NiFe-LDH/FeOOH heterostructure was *in-situ* grown on NF vertically via a facile competing growth strategy of bimetallic ions with OH^- ions, through one-pot hydrothermal treatment at 120 °C for 12 hours in the precursor solution containing H_2O_2 as the oxidizing agent, urea providing OH^- ions, and $Fe(NO_3)_3{\cdot}9H_2O$ as the Ferrum source. After the hydrothermal process, the color of the NF changes from silver gray to brown, indicating the successful growth of the NiFe-LDH/FeOOH heterostructure.

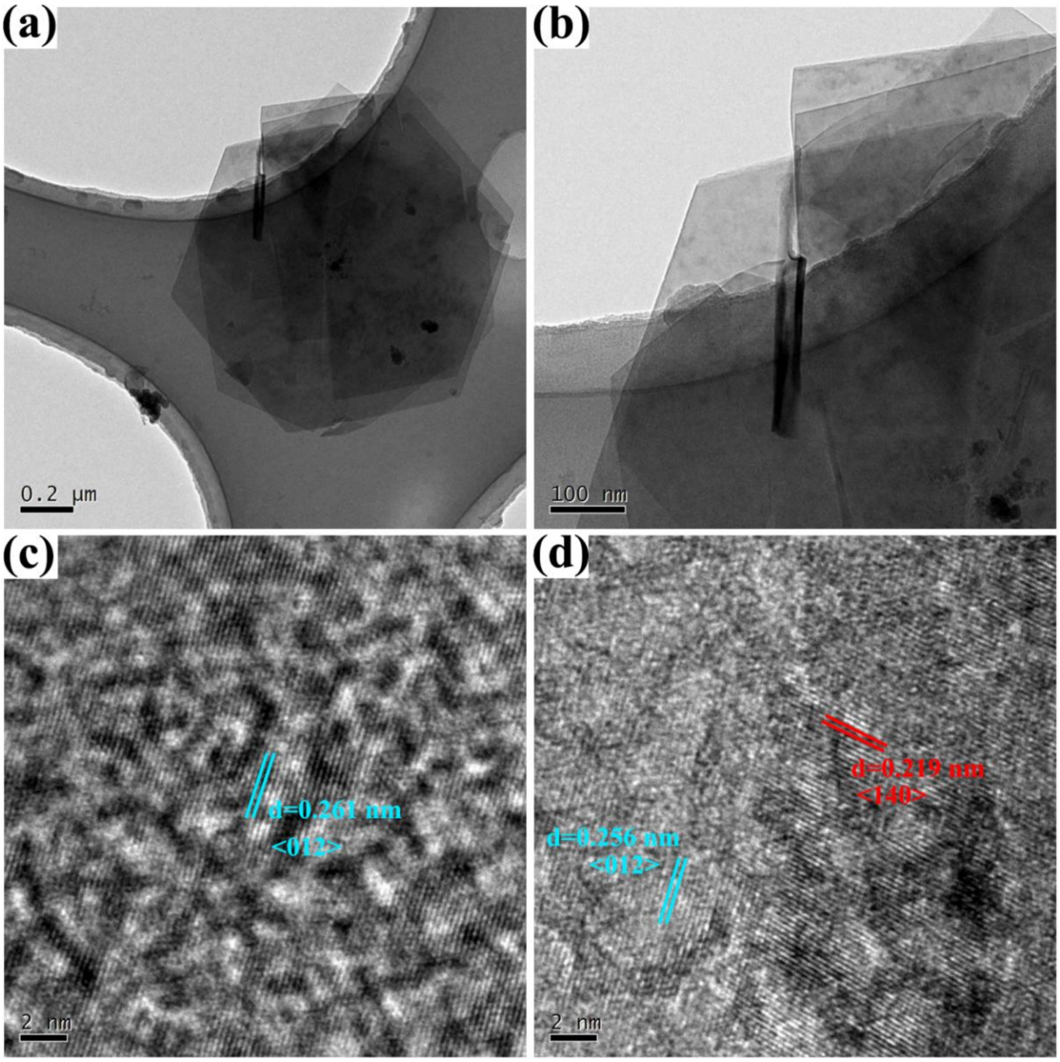

Figure S2. TEM image of NiFe-LDH/FeOOH.

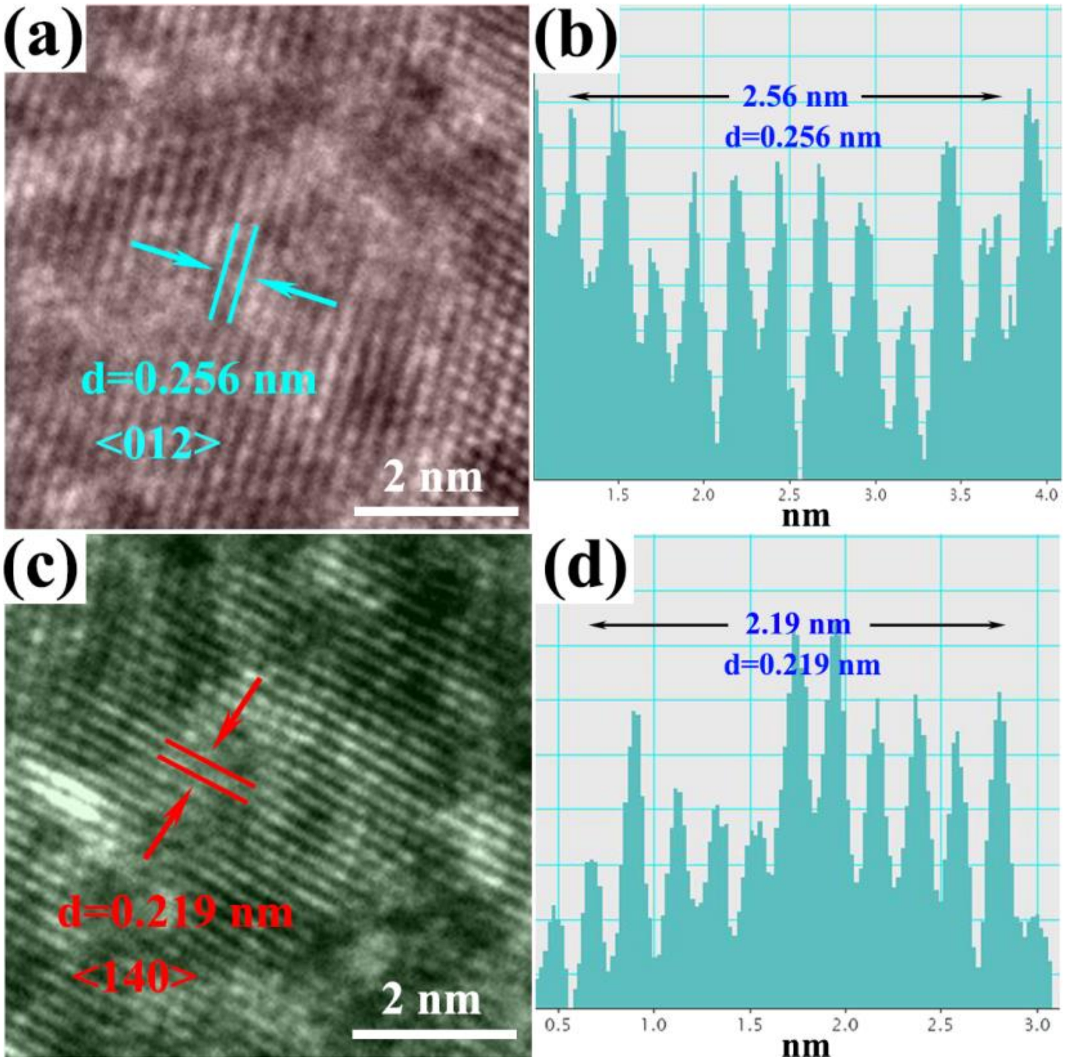

Figure S3. a) Lattice fringe image of NiFe-LDH and b) line scan image indicated by the lattice fringe in (a). c) Lattice fringe image of FeOOH and d) line scan image indicated by the lattice fringe in (c).

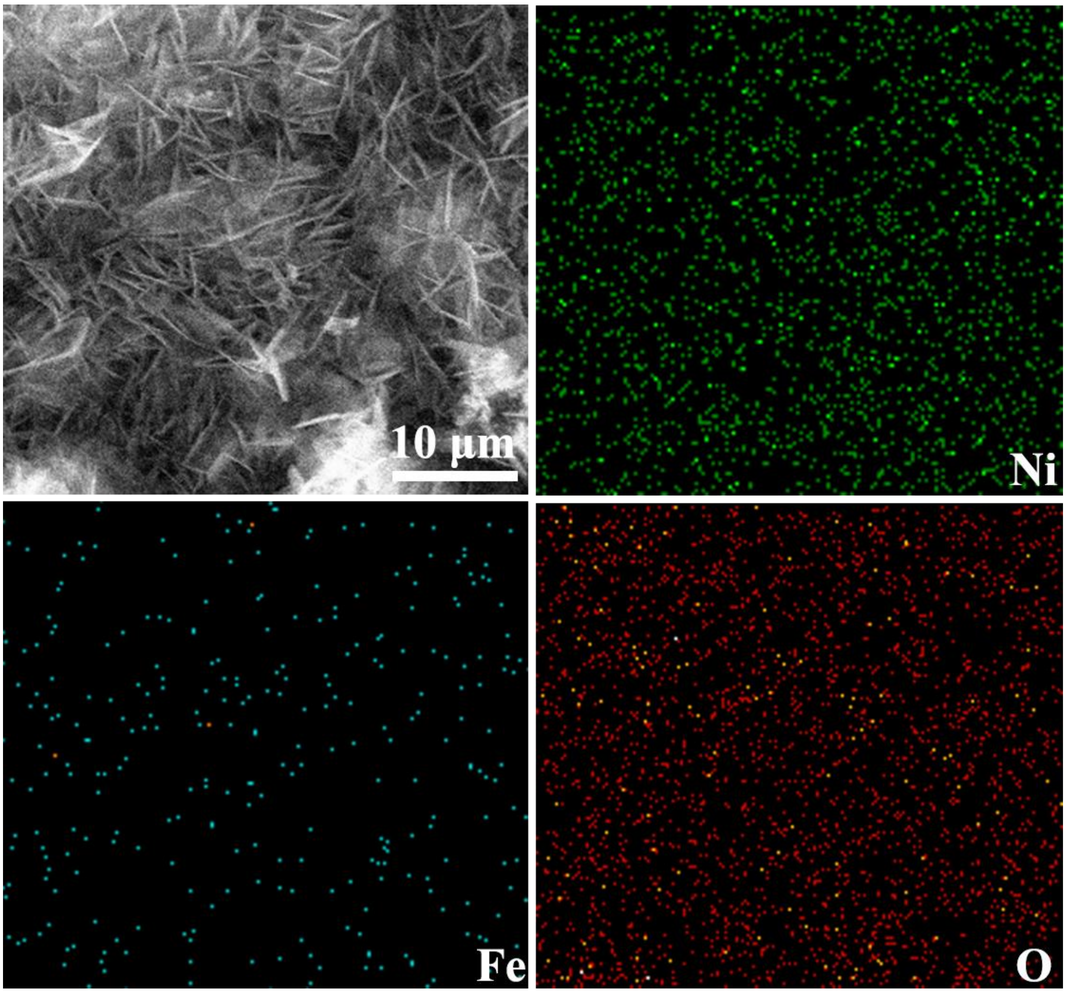

Figure S4. SEM image and corresponding elemental mapping images of NiFe-LDH/FeOOH heterostructures.

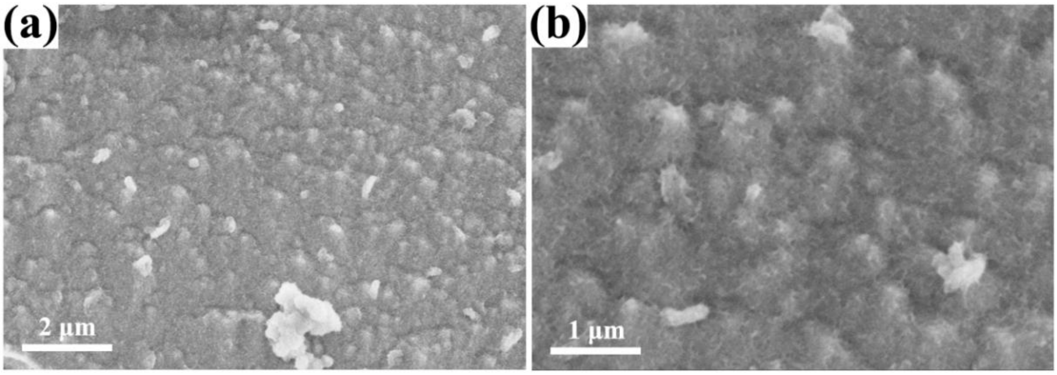

Figure S5. SEM images of $Ni(OH)_2$.

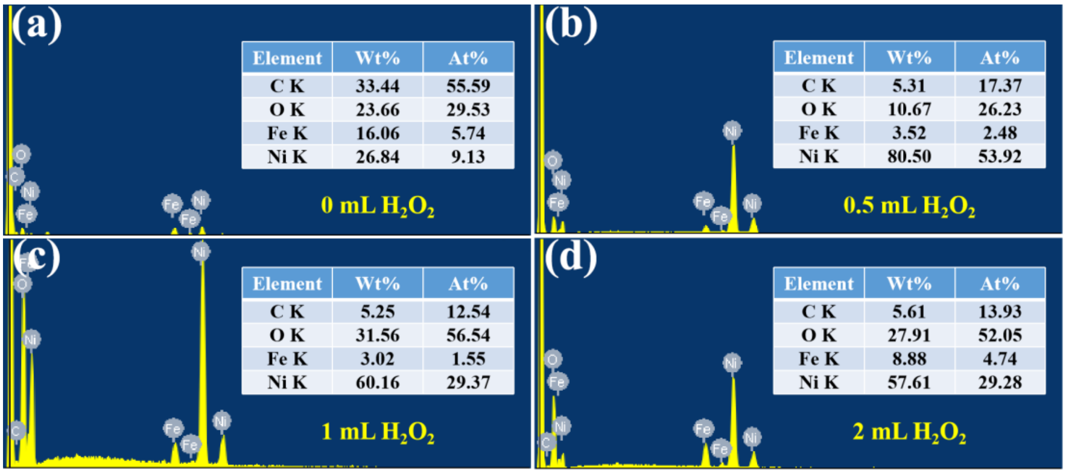

Figure S6. EDS data of samples with a) 0 mL, b) 0.5 mL, c) 1 mL, and d) 2 mL H_2O_2.

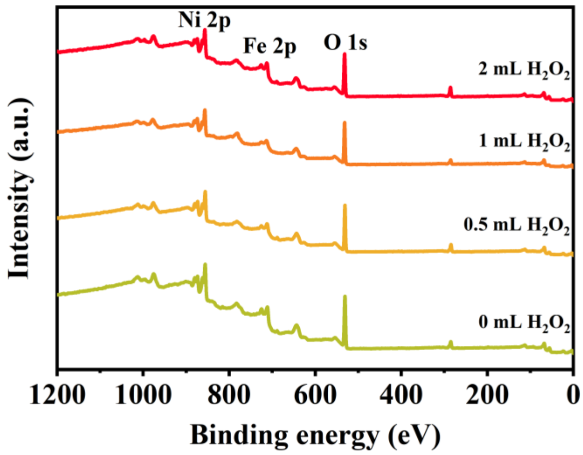

Figure S7. XPS survey of samples with a) 0 mL, b) 0.5 mL, c) 1 mL, and d) 2 mL H_2O_2.

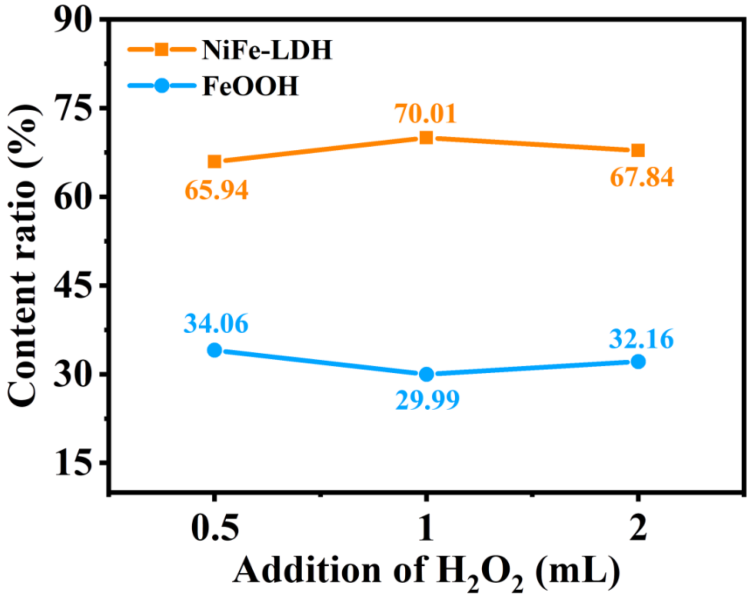

Figure S8. The content ratio of NiFe-LDH and FeOOH in NiFe-LDH/FeOOH heterostructures calculated from XPS characterizes.

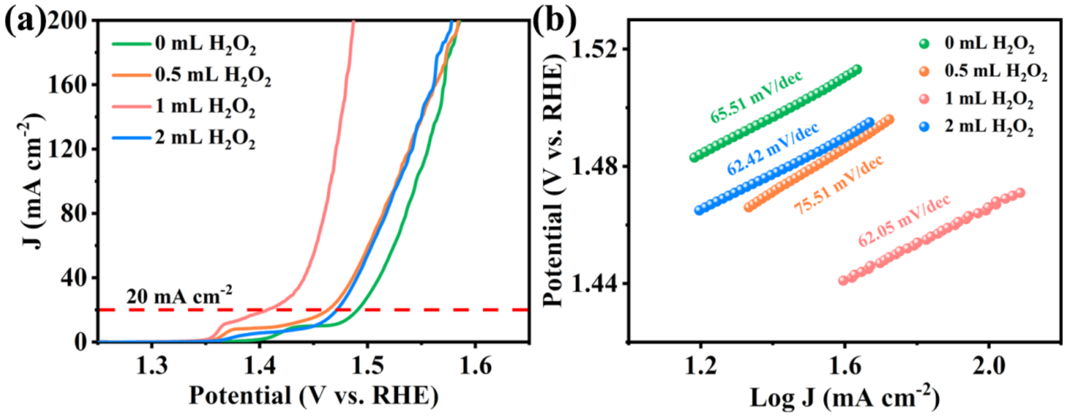

Figure S9. a) LSV curves and b) corresponding Tafel plots of samples synthesized with different H_2O_2 addition in 1.0 M KOH.

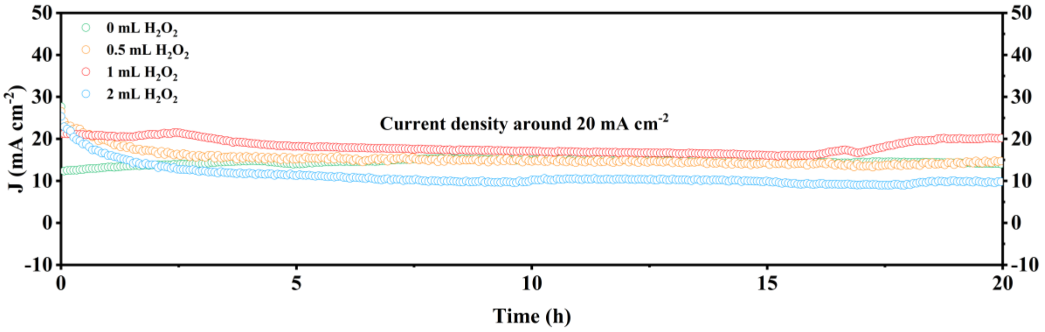

Figure S10. Time-dependent OER performance of the samples.

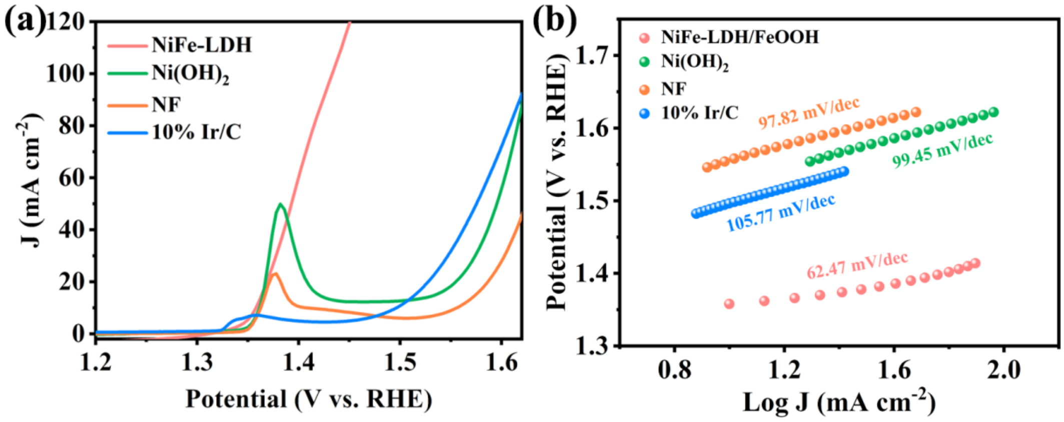

Figure S11. a) SCV curves and b) corresponding Tafel plots of NiFe-LDH/FeOOH, $Ni(OH)_2$, NF, and 10% Ir/C electrodes.

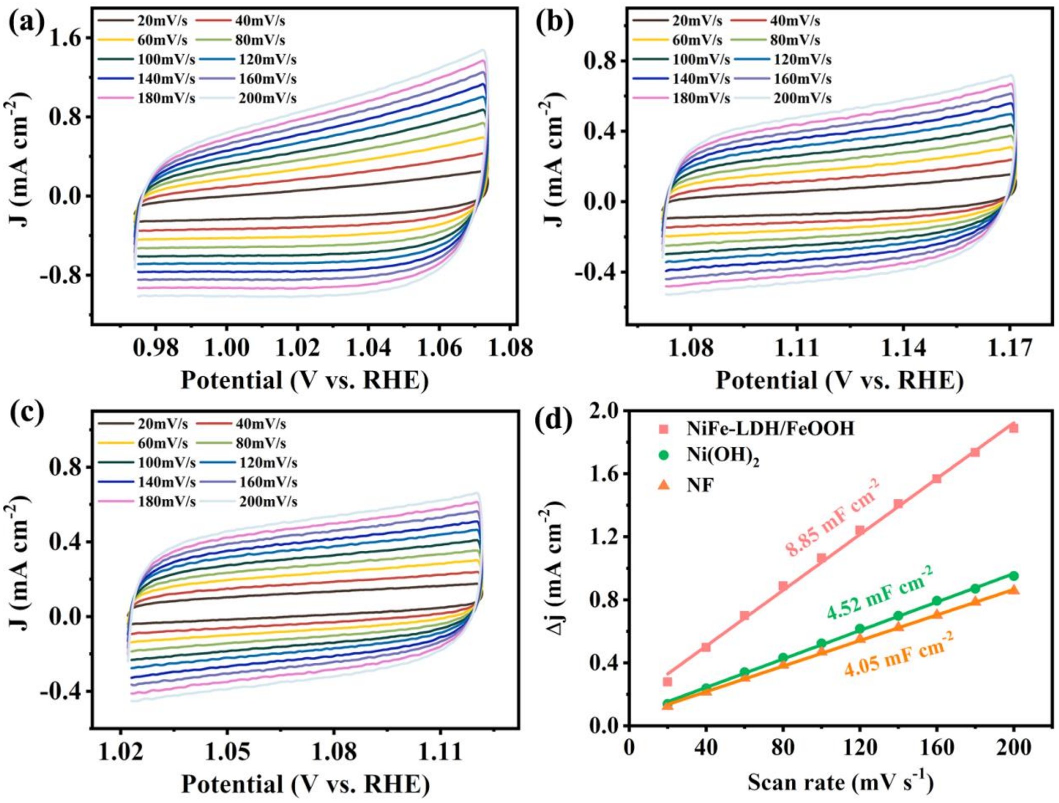

Figure S12. Cyclic voltammograms (CVs) in 1.0 M KOH for OER. a) NiFe-LDH/FeOOH, b) $Ni(OH)_2$ and c) NF in the non-Faradaic capacitance current range at scan rates of 20, 40, 60, 80, 100, 120, 140, 160, 180, and 200 mV s^{-1}. d) The double-layer capacitance (C_{dl}) for NiFe-LDH/FeOOH, $Ni(OH)_2$, and NF electrodes derived from the CV plots.

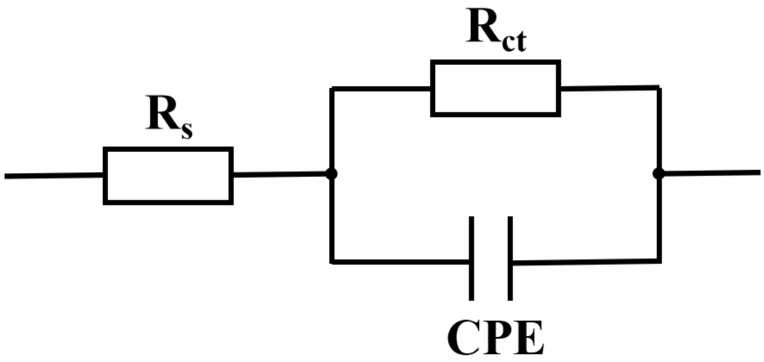

Figure S13. An equivalent circuit for the electrode/electrolyte interface.

The Nyquist curves can be fitted into a simple equivalent circuit consisting of a solution resistance (Rs) element, a charge transfer resistance (Rct) element, and a constant phase angle element (CPE) connected in parallel.

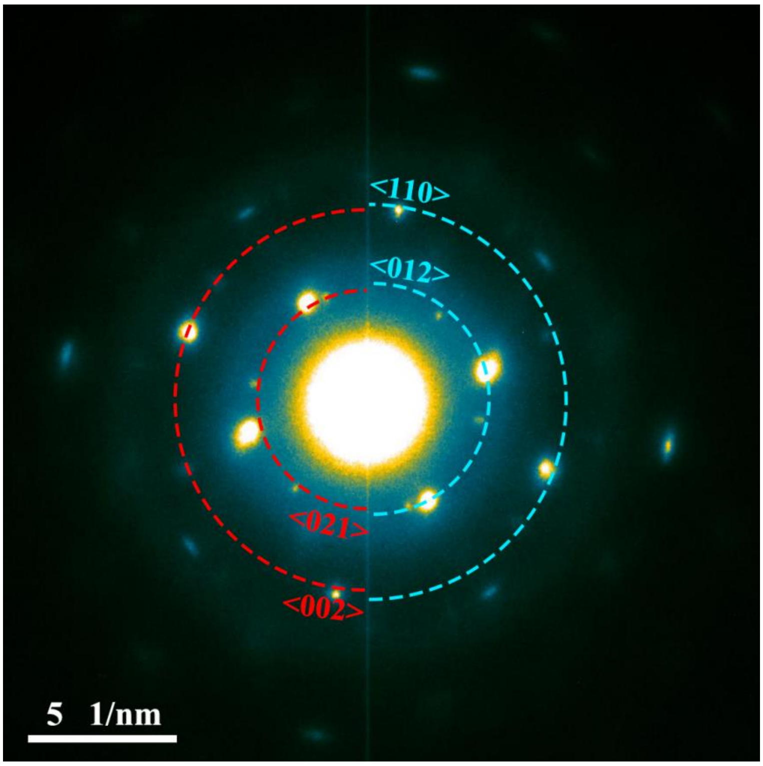

Figure S14. SAED image of NiFe-LDH/FeOOH heterostructure after stability test.

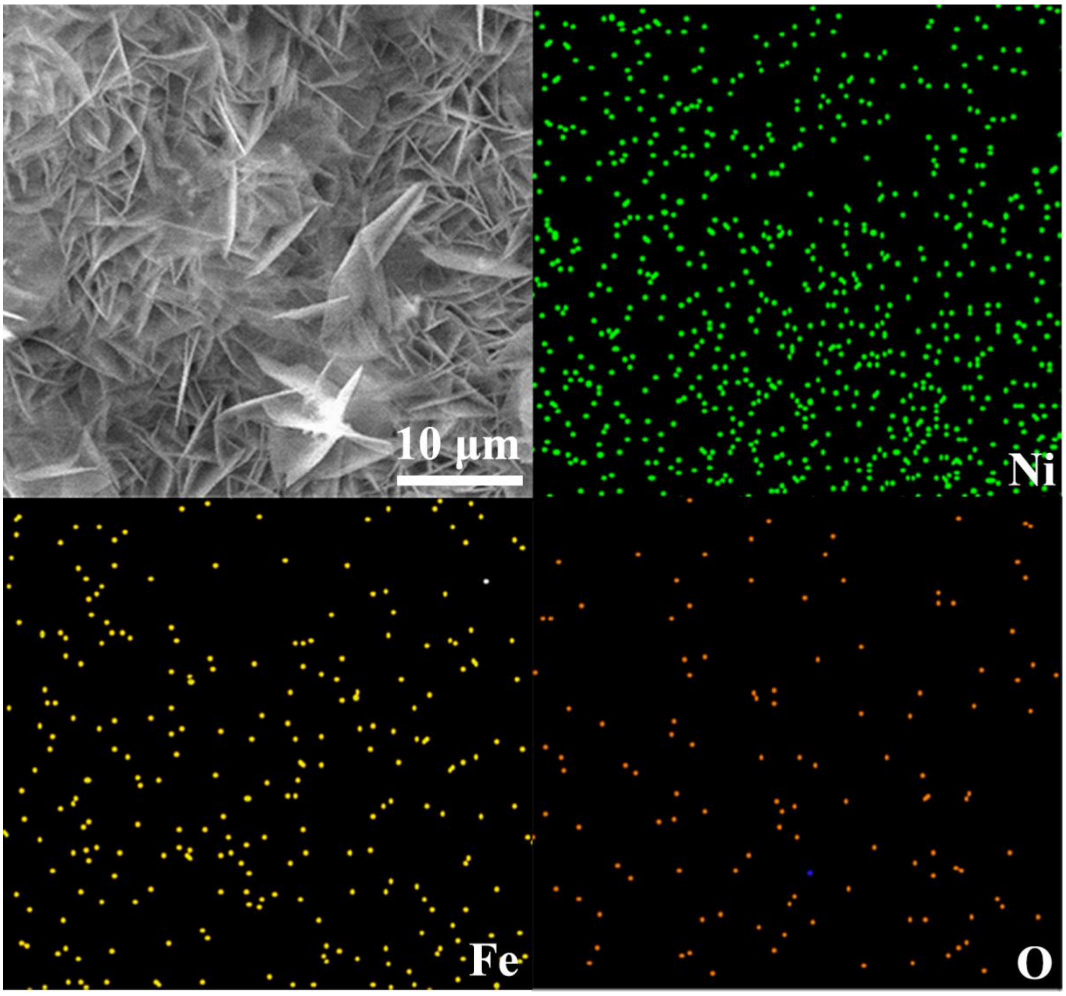

Figure S15. SEM image and corrsponding elemental mapping images of NiFe-LDH/FeOOH heterostructure after stability test.

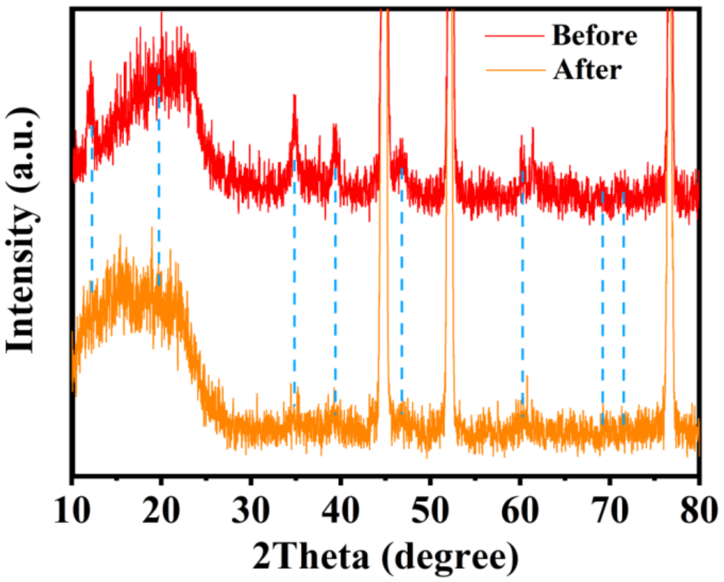

Figure S16. XRD patterns of NiFe-LDH/FeOOH before and after OER.

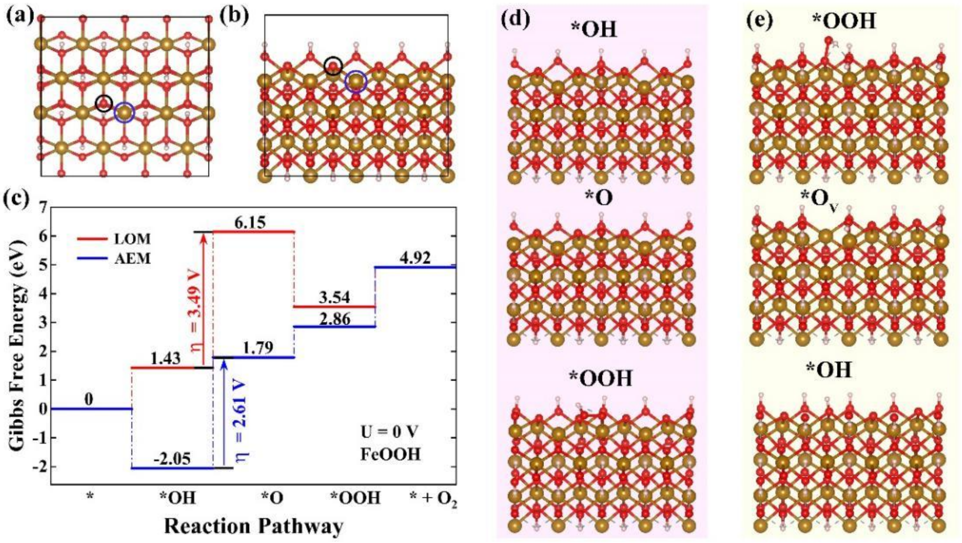

Figure S17. The a) top and b) side view of FeOOH. The Fe, O, and H atoms are represented by brown, red, and pink balls, respectively. The active sites for AEM and LOM are highlighted by blue and black circles, respectively. c) The Gibbs free energy diagram of AEM and LOM pathways on FeOOH, where the arrows show the RDS and η are corresponding overpotentials. The adsorption intermediates of d) AEM pathways (*OH, *O and *OOH) and e) LOM pathways (*OOH, * O_V and *OH) for FeOOH.

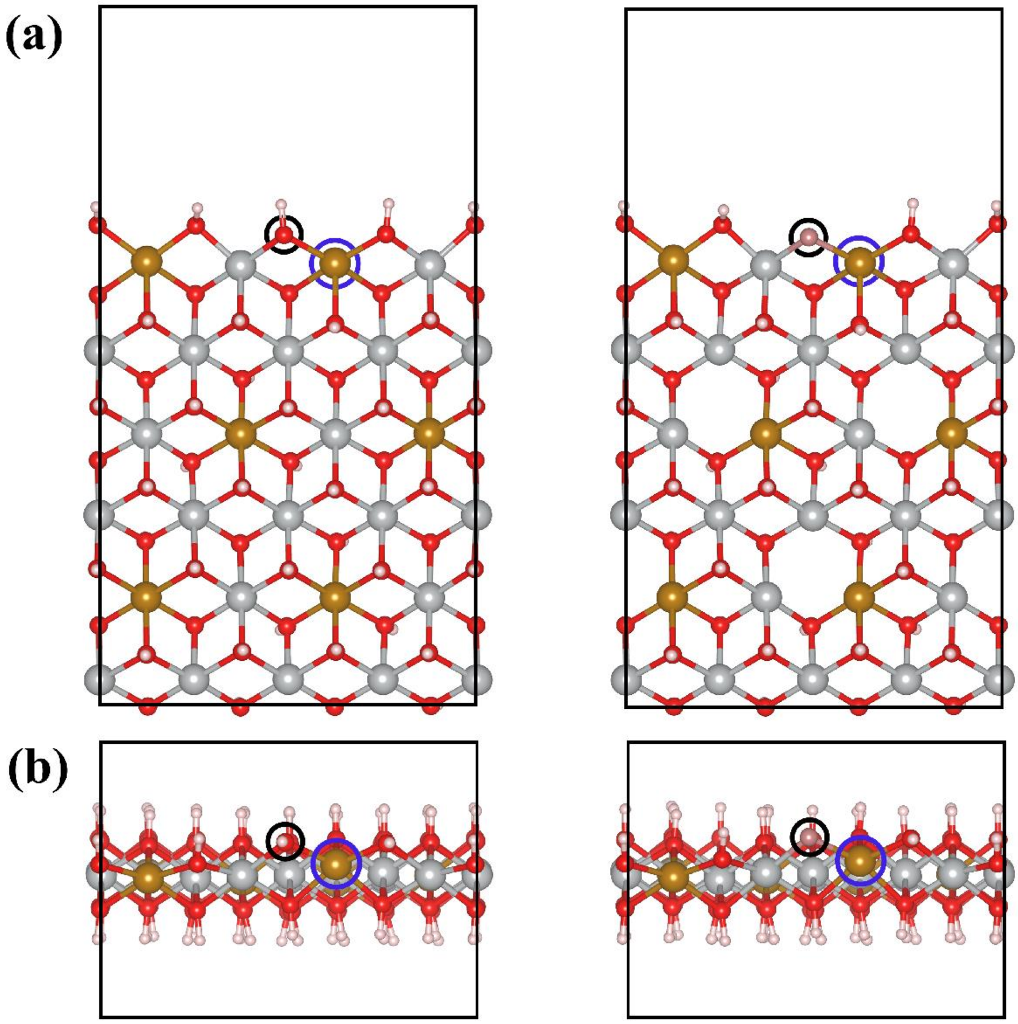

Figure S18. The a) top and b) side view of NiFe-LDH without and with oxygen vacancies. The Ni, Fe, O, and H atoms are represented by gray, brown, red, and pink balls, respectively. The active sites for AEM and LOM are highlighted by blue and black circles, respectively.

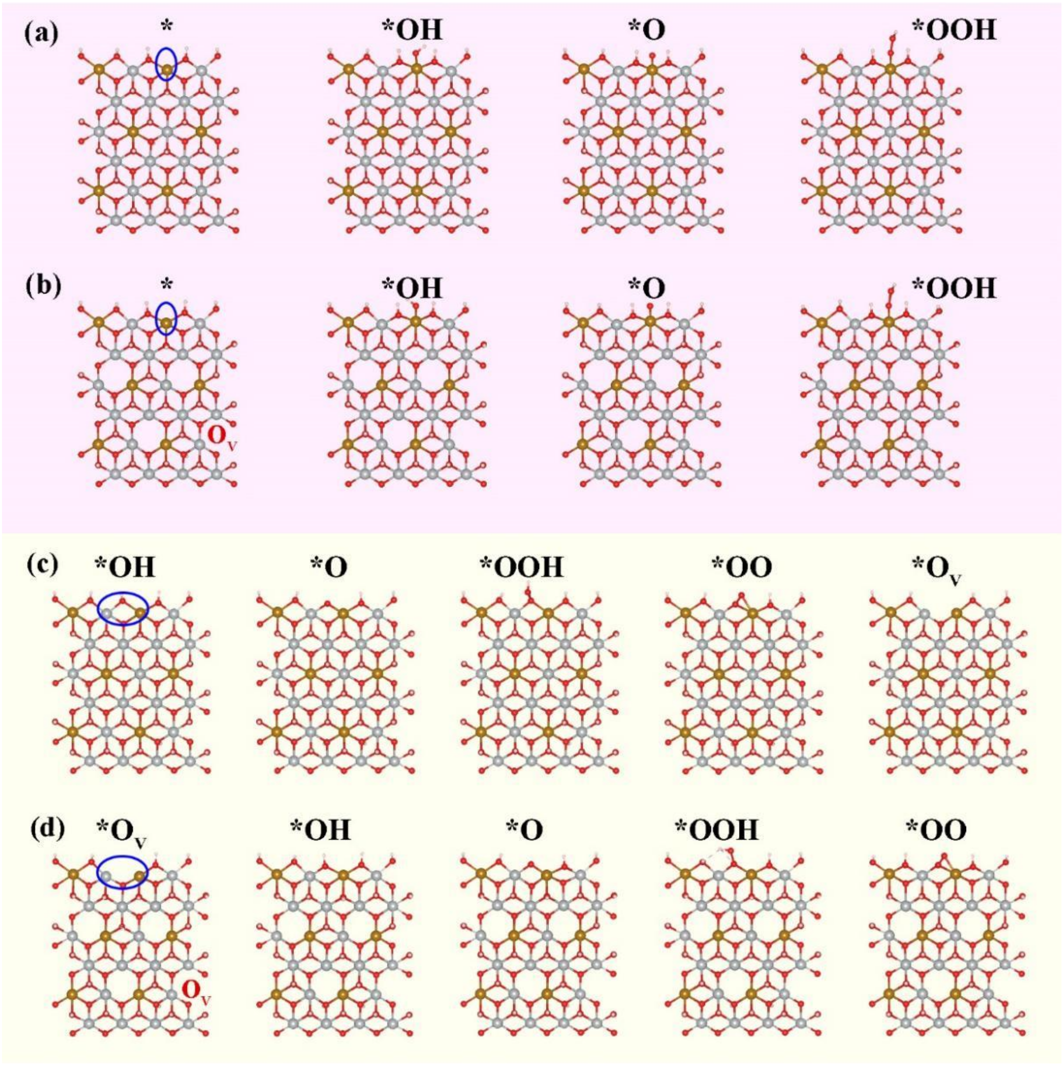

Figure S19. The slab structures and adsorption intermediates (*OH, *O and *OOH) of AEM pathways for NiFe-LDH a) without O_V and b) with O_V. The adsorption sites are highlighted by blue circles. The slab structures and adsorption intermediates (*OH, *O, *OOH and *OO) of LOM pathways for NiFe-LDH c) without O_V and d) with O_V. The circled sites show the active lattice oxygen or active oxygen vacancy.

Table S1. Comparison of electrocatalytic OER performances of NiFe-LDH/FeOOH with other reported catalysts in 1.0 M KOH. η_{100} corresponds to the overpotential at current density of 100 mA cm^{-2}. (Note: *: Value calculated from curves shown in the respective reference. NF: nickel foam.)

Catalyst	η_{100} (mV)	Substrate	Reference
**NiFe-LDH/FeOOH**	**236**	**NF**	**This work**
FeOOH/NiFe-LDH	238	NF	J. Mater. Chem. A, 2021, 9, 21785–21791.
NiFe LDH-PMo12	249	NF	Adv. Mater. 2022, 34, 2110696.
F-NiFe-A	252	NF	Nano Lett., 2021, 21, 492-499.
$Ni_{0.3}Fe_{0.7}$-LDH	256	NF	Appl. Catal. B: Environ., 2023, 323, 122091.
Ni_2P-Fe_2P/NF	261	NF	Adv. Funct. Mater., 2021, 31, 2006484.
NiMoN@NiFeN	277	NF	Nat. Commun., 2019, 10, 5106.
Ni(Fe)OOH-FeS_x	300	NF	Nat. Commun., 2020, 11, 5075.
NiFeCr/NF	320	NF	Energy Environ. Sci., 2020, 13, 4225.
Te, Cl-NiFe MOF	245*	NF	Chem. Eng. J., 2022, 439, 135720.
Nb-NiFe-LDH	260*	NF	Chem. Eng. J., 2022, 427, 131643.
S-NiFe LDH	270*	NF	Adv. Energy Mater. 2022, 32, 2202522.
FeOOH/$Ni(OH)_2$	280*	NF	Nanoscale, 2020, 12, 983-990.
Fe-β-$Ni(OH)_2$	280*	NF	ACS Energy Lett., 2019, 4, 622-628.

References

[1] M. Kuang, Y. Wang, W. Fang, H. Tan, M. Chen, J. Yao, C. Liu, J. Xu, K. Zhou, Q. Yan, *Adv. Mater.* **2020**, 32, 2002189.

[2] G. Kresse, J. Hafner, *Phys. Rev. B* **1994**, 49, 14251.

[3] G. Kresse, D. Joubert, *Phys. Rev. B* **1999**, 59, 1758.

[4] P. E. Blöchl, *Phys. Rev. B* **1994**, 50, 17953.

[5] J. P. Perdew, K. Burke, M. Ernzerhof, *Phys. Rev. Lett.* **1996**, 77, 3865.

[6] J. A. White, D. M. Bird, *Phys. Rev. B* **1994**, 50, 4954.

[7] T. Bučko, J. Hafner, S. Lebègue, J. G. Ángyán, *J. Phys. Chem. A* **2010**, 114, 11814.

[8] S. Froyen, *Phys. Rev. B* **1989**, 39, 3168.

[9] F. Dionigi, Z. H. Zeng, I. Sinev, T. Merzdorf, S. Deshpande, M. B. Lopez, S. Kunze, I. Zegkinoglou, H. Sarodnik, D. X. Fan, A. Bergmann, J. Drnec, J. F. de Araujo, M. Gliech, D. Teschner, J. Zhu, W. X. Li, J. Greeley, B. Roldan Cuenya, P. Strasser, *Nat. Commun.* **2020**, 11, 2522.
